# N4-hydroxycytidine, the active compound of Molnupiravir, promotes SARS-CoV-2 mutagenesis and escape from a neutralizing nanobody

**DOI:** 10.1016/j.isci.2023.107786

**Published:** 2023-08-30

**Authors:** Arne Zibat, Xiaoxiao Zhang, Antje Dickmanns, Kim M. Stegmann, Adrian W. Dobbelstein, Halima Alachram, Rebecca Soliwoda, Gabriela Salinas, Uwe Groß, Dirk Görlich, Maik Kschischo, Bernd Wollnik, Matthias Dobbelstein

**Affiliations:** 1 Department of Human Genetics, University Medical Center Göttingen, 37073 Göttingen, Germany; 2 Department of Mathematics and Technology, University of Applied Sciences Koblenz, 53424 Remagen, Germany; 3 Department of Informatics, Technical University of Munich, 81675 Munich, Germany; 4 Department of Molecular Oncology, Göttingen Center of Molecular Biosciences (GZMB), University Medical Center Göttingen, 37077 Göttingen, Germany; 5 Max Planck Institute for Biology, 72076 Tübingen, Germany; 6 NGS Integrative Genomics Core Unit, Department of Human Genetics, University Medical Center Göttingen, 37077 Göttingen, Germany; 7 Department of Medical Microbiology and Virology, University Medical Center Göttingen, 37075 Göttingen, Germany; 8 Max Planck Institute for Multidisciplinary Sciences, 37077 Göttingen, Germany; 9 Cluster of Excellence "Multiscale Bioimaging: from Molecular Machines to Networks of Excitable Cells" (MBExC), University of Göttingen, 37075 Göttingen, Germany

**Keywords:** Pharmaceutical science, Immunology, Virology, Structural biology

## Abstract

N4-hydroxycytidine (NHC), the active compound of the drug Molnupiravir, is incorporated into SARS-CoV-2 RNA, causing false base pairing. The desired result is an “error catastrophe,” but this bears the risk of mutated virus progeny. To address this experimentally, we propagated the initial SARS-CoV-2 strain in the presence of NHC. Deep sequencing revealed numerous NHC-induced mutations and host-cell-adapted virus variants. The presence of the neutralizing nanobody Re5D06 selected for immune escape mutations, in particular p.E484K and p.F490S, which are key mutations of the Beta/Gamma and Omicron-XBB strains, respectively. With NHC treatment, nanobody resistance occurred two passages earlier than without. Thus, within the limitations of this purely *in vitro* study, we conclude that the combined action of Molnupiravir and a spike-neutralizing antagonist leads to the rapid emergence of escape mutants. We propose caution use and supervision when using Molnupiravir, especially when patients are still at risk of spreading virus.

## Highlights

• Molnupiravir might give rise to new SARS-CoV-2 variants when used without precautions
• *In vitro*, the active compound of Molnupiravir gives rise to nanobody-resistant variants
• We provide an integrated view on the selection of nanobody-escape mutants

## Graphical abstract

**Figure:**
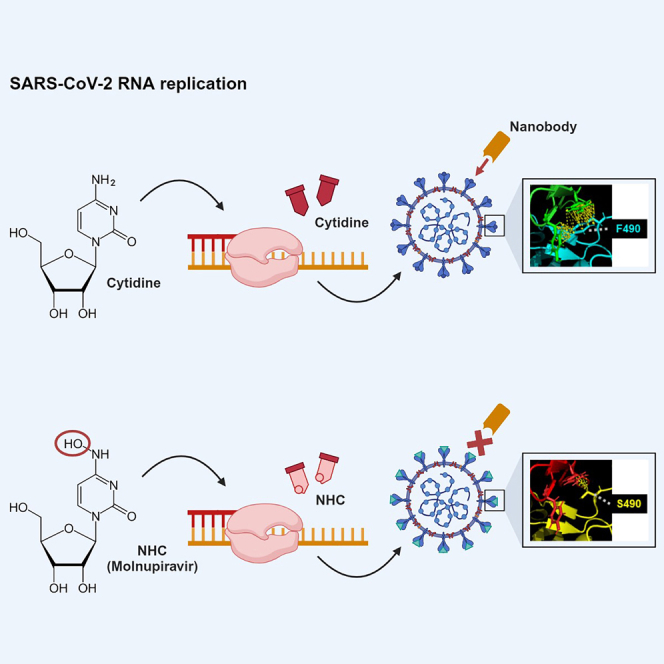

## Introduction

The COVID-19 pandemic has killed more than six million people and still gives rise to thousands of deaths weekly worldwide. Besides an unprecedented vaccination effort, this also raises the urgent need for effective therapies.^1^ Therapeutic antibodies interfere with virus entry to host cells, but they are expensive and require intravenous infusion, making it difficult to use them broadly. Furthermore, new virus variants such as Omicron often resist such antibodies.^2^ On the other hand, some orally available small molecule-drugs directly interfere with the replication of SARS-CoV-2. Currently, such antivirals approved for treating COVID-19 are either targeting the cleavage of viral peptides or otherwise RNA replication. Of note, a nucleoside analogue named Molnupiravir yielded a favorable outcome in clinical trials. Molnupiravir suppressed the virus below detectability in COVID-19 patients.^3^ When Molnupiravir was provided to patients early after the onset of COVID-19 symptoms, it also reduced the likelihood of hospitalization by roughly 30%.^4^ A more recent study found a lower risk of death and in-hospital disease progression when using Molnupiravir,^5^^,^^6^ although other studies were unable to confirm improvements in hospitalization.^7^^,^^8^^,^^9^ Based on such results, Molnupiravir was approved for COVID-19 treatment in the United Kingdom of Great Britain^10^ and Israel,^11^ and for emergency use in the European Union and the USA.

Molnupiravir is orally applicable. Upon resorption and cleavage of an ester bond, the active compound is released, i.e., β-*d*-N4-hydroxycytidine (NHC).^12^^,^^13^^,^^14^^,^^15^ Thus, Molnupiravir is a pro-drug of the ribonucleoside analogue NHC. In comparison to cytidine, NHC has the same structure but carries a hydroxylated amino group (nitrogen 4) at the pyrimidine base. When an infected cell takes up NHC, the molecule undergoes triple phosphorylation to obtain a hydroxylated CTP analogue (NHCTP), which can now become a substrate to the viral RNA-dependent RNA polymerase (RdRp). Unlike other antiviral nucleoside analogues, NHC and its metabolites do not inhibit the progression of RdRp.^16^^,^^17^ Instead, NHC becomes incorporated into the nascent viral RNA, with continued RNA synthesis thereafter. Several NHC molecules can thus become part of a nascent viral RNA genome. The antiviral effect manifests itself when RNA replication continues, e.g., when using a minus RNA strand to synthesize a new plus strand virus genome, or vice versa. Due to a tautomeric interconversion within the NHC base, NHC can not only form a base pair with guanine (as cytidine does) but also with adenine. Thus, the incorporation of NHC gives rise to multiple mutations within the virus genome.^18^^,^^19^ When frequent enough, these mutations disable the synthesis of functional virus proteins, especially during subsequent rounds of infection. This outcome, termed ‘error catastrophe’, likely causes the therapeutic suppression of virus replication. Molnupiravir was also found effective against the Omicron variant of SARS-CoV-2 in an animal model.^20^ Moreover, our recent work strongly suggests that the combination of Molnupiravir with inhibitors of endogenous pyrimidine synthesis can further enhance this desirable therapeutic effect, at least in experimental systems, presumably by enhancing the incorporation of NHC into virus RNA.^21^

Despite these impressive developments, a drug that causes virus mutations cannot be without concern, and the approval of Molnupiravir for clinical use was heavily debated. One reservation consisted of the possibility that Molnupiravir might also induce mutations in the cellular genome, which would then cause damage to embryos (in case of pregnancy) or perhaps contribute to malignant transformation.^22^^,^^23^^,^^24^^,^^25^

Another concern, however, was raised more recently: Could Molnupiravir treatment facilitate the occurrence of mutant virus with increased virulence^26^^,^^27^^,^^28^^,^^29^^,^^30^^,^^31^? Here, the possibility of raising viable, mutant viruses is at the center. When handling the drug properly, specific guidelines indicate dose and time of administration. However, when treating patients with insufficient doses of Molnupiravir, or when ending the treatment too early, this might leave a population of viruses that carry mutations but are still replication-competent. From such a pool, the fittest mutants might not only propagate within the body of the same patient but also spread further through the population. Fitness of such induced virus mutants might include immune evasion (especially when a population was vaccinated before) but also increased capabilities of transmission and further replication. However, there is little if any available evidence whether such a scenario might actually occur, neither in Molnupiravir-treated COVID-19 patients nor in experimental systems.

In the present study, we tried to model Molnupiravir-induced virus mutagenesis *in vitro*, recapitulating the treatment of SARS-CoV-2 infection by incubating infected cultured cells with the active compound of Molnupiravir, NHC. The virus population released from such cells indeed contained multiple mutations, as revealed by deep sequencing. Strikingly, two to four passages of this virus pool gave rise to selection of distinct mutants with apparent gains of fitness. The Furin cleavage site within the spike protein was mutated, plausibly contributing to virus replication in Vero cells. Most impressively, however, the rise of immune-escape mutants was recapitulated in this system. When incubating the pool with a potently neutralizing nanobody, resistant virus mutants were rapidly selected that had reproducibly mutated specific residues in the spike protein, most notably p.F490S, p.E484K, or p.G446D. These residues, according to structure analyses and AlphaFold predictions, were major mediators of the spike-nanobody interaction. Hence, at least in an *in vitro* system, NHC can indeed give rise to virus pools containing readily selectable mutants, with efficient adaptation to the cellular environment and complete resistance to a neutralizing nanobody.

## Results

### Replicating SARS-CoV-2 in the presence of NHC gives rise to a mutant virus pool from which nanobody-resistant viruses were selected

**Figure 1:**
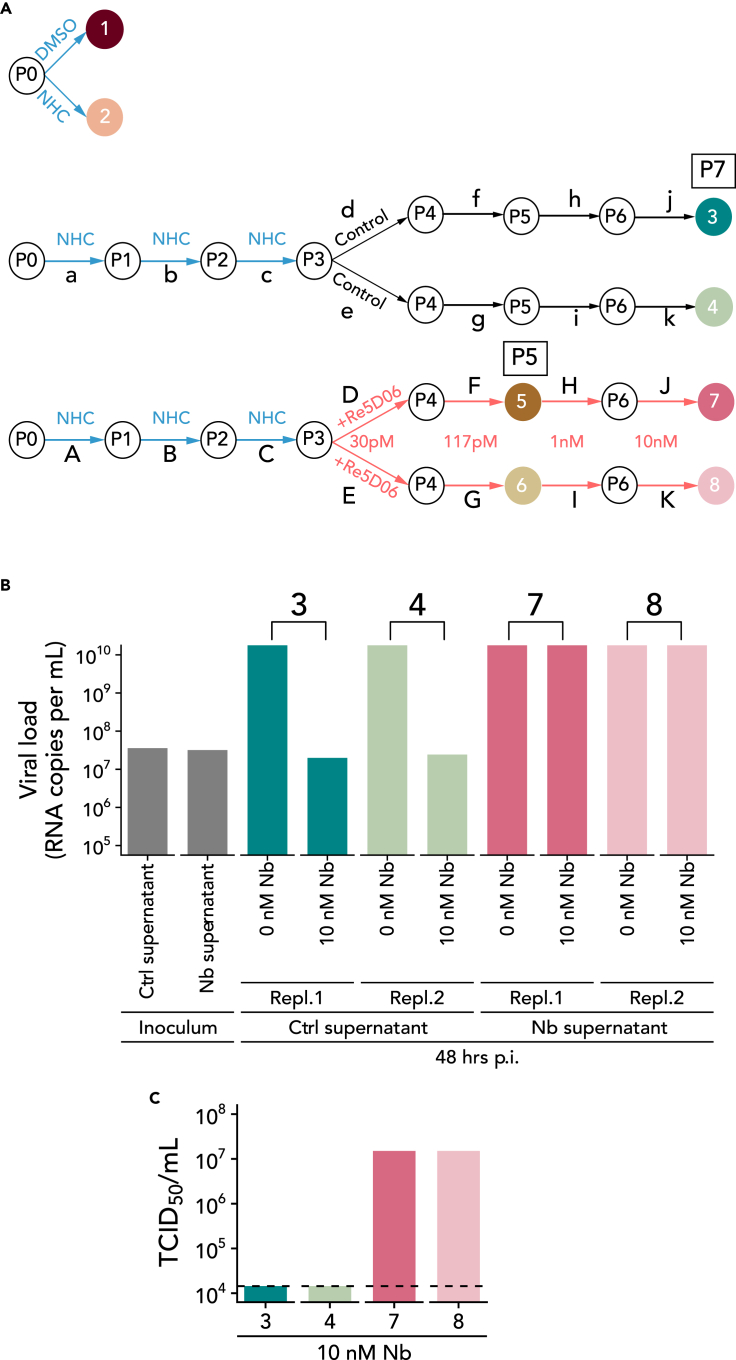
SARS-CoV-2 propagation upon incubation with N4-hydroxycytidine (NHC) and selection of nanobody-resistant virus populations (A) Virus propagation. Vero E6 cells were treated with 300 nM N4-hydroxycytidine (NHC) and subsequently infected with SARS-CoV-2, strain Göttingen.^35^ The NHC concentration was chosen to leave a detectable cytopathic effect (CPE), indicating virus replication. Virus RNA was isolated for sequencing without (#1) or with (#2) NHC treatment. In another set of experiments, virus was passaged in the presence of NHC thrice (300–400 nM, cf. Table S1) and then subjected to parallel passaging either in plain media, or while adding the potently neutralizing nanobody Re5D06^34^ in the indicated concentrations, again chosen to leave a CPE. The virus samples indicated by numbers were subjected to RNA isolation and deep sequencing analysis with high accuracy (paired-end sequencing). The samples were taken after the virus was passaged four times in non-treated cells (#3 and #4), or passaged two times (#5 and #6) or four times (#7 and #8) in the presence of the nanobody. Concentrations of virus RNA in the inocula and supernatants, as well as drug concentrations, are indicated for each step in Table S1. (B) Acquired resistance toward nanobody Re5D06. The indicated virus pools were used to infect a fresh monolayer of Vero E6 cells, in the presence or absence of nanobody Re5D06 at a concentration of 10 nM, which is more than 100 times the neutralizing concentration for the original SARS-CoV-2.^34^ At 48 h p.i., virus RNA in the supernatant was quantified by RT-PCR to reflect the capability of the virus to replicate. The virus populations that had been obtained by passaging in the presence of the nanobody (#7, #8) were capable of replicating efficiently even when the nanobody was added. In contrast, the virus populations obtained at the same number of passages in plain media (#3, #4) were now still neutralized by the nanobody, reflected by the absence of detectable virus replication when the nanobody was added to the virus inoculum. (C) Resistance toward nanobody Re5D06, as revealed by quantifying infectious units. The virus-containing supernatant of (B) was titrated on 96-well-plates to determine the Median Tissue Culture Infectious Dose (TCID_50_, n = 4). Data of two technical replicates are represented as mean. Fluorescence signals were detected by automated microscopy (see Figure S1 for fluorescence images). The dashed line indicates the TCID_50_ detection limit 1.44 × 10^4^/mL.

Vero E6 cells replicate SARS-CoV-2 with high efficiency^32^^,^^33^ and were therefore chosen to carry out this study. We pre-treated Vero E6 cells with NHC at a concentration that we had previously determined to reduce virus yield 5- to 10-fold, thus leaving enough infectious particles for subsequent passages of virus. For selection experiments (Figure 1A), the virus was first passaged through NHC-treated cells three times and then in plain media for four more passages. In a parallel experiment, the virus obtained from NHC-treated cells was passaged in the presence of increasing amounts of a neutralizing nanobody, Re5D06. We have previously described Re5D06 as an extremely potent virus antagonist, capable of neutralizing even at 2-digit picomolar concentrations.^34^ To model the presence of a neutralizing antibody in a patient, we chose Re5D06 because it is the best-characterized nanobody of our previous study. Each passaging or selection experiment was carried out in parallel with two samples. In each passage, we controlled the content of viral genomic RNA to define the inoculum of the subsequent passage (Table S1).

During the passage of virus pools in the presence of nanobody, we noticed that the tolerance of the virus toward the nanobody increased. Whereas the initial virus pool was largely neutralized by the nanobody, even 10 nM of the nanobody no longer prevented infection with the virus pool obtained at the end of the selection procedure. We observed this by quantifying both the virus RNA (Figure 1B) and the infectious particles (Figures 1C and S1) in the supernatant of the cells. This was the first evidence that treatment with the active compound of Molnupiravir had given rise to selectable resistance of virus mutants.

### NHC induces multiple transition mutations in replicating SARS-CoV-2, and further passaging selects a subset of specific mutants

**Figure 2:**
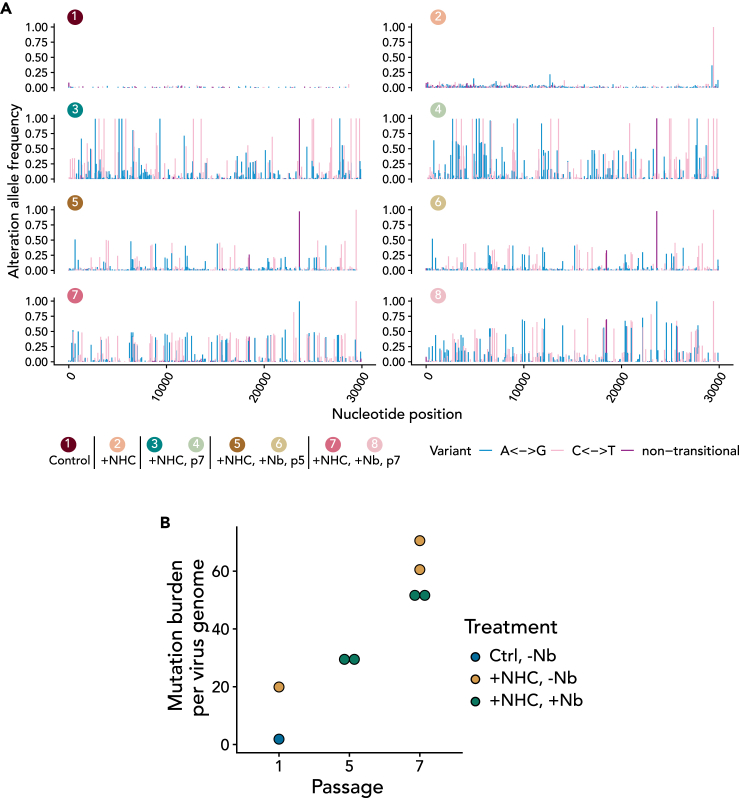
Accumulation of high-frequency mutations within the NHC-exposed virus population after further passaging (A) Frequency and position of mutations observed in ≥1% of the reads covering the mutated site. The number of reads containing the mutation was divided by the sum of reference read counts and alteration read counts. These numbers represent the percentage of each viral population that carries the respective mutation. The frequencies of all these mutations are plotted for each virus population, as designated by numbers and colors in Figure 1A. Note that the vast majority of mutations were transitions between purines (blue) or pyrimidines (pink) rather than transversions (violet), and that the frequency and number of detectable mutations increased after passaging the virus in the presence of NHC. Different patterns were observed between virus populations that were passaged in the presence versus the absence of nanobody. All details of the mutations are outlined in Table S2. (B) Mutation burden per virus genome of the virus pools described in (A). This is the estimated average number of mutations within each virus genome within the pool. Note that NHC treatment increased the mutation burden compared to the control-treated virus pool, and that the mutation burden further increased by passaging in the presence or absence of nanobody. In the case of passage 5, we only sequenced the nanobody-selected samples, to get a better idea of how resistance to the nanobody evolved.

Before and after the incubation with NHC, we subjected the pool of virus RNA to paired-end RNA sequencing analysis (Figure 2A; Tables S2 and S3). As expected, the RNA pool from the supernatant of NHC-treated cells contained many more mutations than the initial virus inoculum that we had described previously,^35^ albeit most of them at a frequency of less than 10% each. The few mutations that occurred in the absence of NHC were asymmetric, with C→U (or T when sequencing cDNA) occurring more often than U→C (Figure S2), in agreement with a previous report describing the same asymmetry in naturally occurring mutations.^36^ NHC increased the frequency of transitions between pyrimidines or between purines but not transversions between purines and pyrimidines (Figure S2). This corresponds to the mechanism by which NHC induces mutations.^16^^,^^37^ The mutation burden per virus genome was higher after passaging SARS-CoV-2 in the presence of NHC compared to a control-treated virus pool (Figure 2B). With further passaging, some of these mutations gained frequency, i.e., they were found at a greater proportion of all sequencing reads that comprised the same position in the virus genome.

### Passaging of an NHC-induced pool of virus mutants limits stop-gain mutations to non-essential ORFs

**Figure 3:**
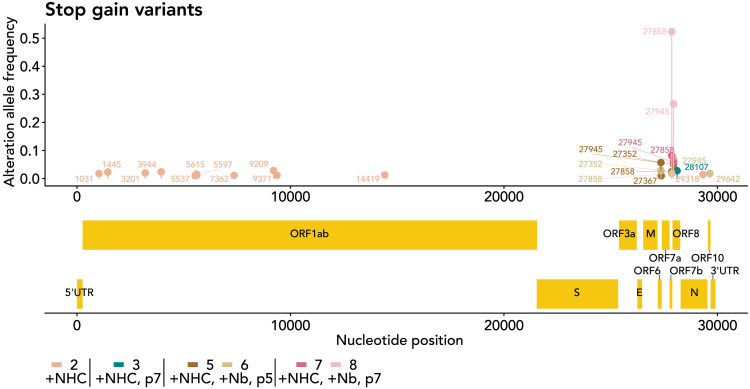
Elimination of stop-gain variants from most parts of the virus genome upon passaging Mutations that led to a novel stop codon within coding regions of the virus (stop-gain variants, nonsense mutations) were filtered from the sequencing data shown in Figure 2A and Table S2, and displayed on the diagram. The initial virus population (#1) did not contain such mutations at detectable levels, not even in regions considered non-essential. Passaging the virus in NHC-treated cells (#2) yielded a number of such mutations across the virus genome; mutations with a frequency ≥1% are indicated. Upon passaging the virus pool with or without nanobody, most of the virus genome was depleted of such mutations, except for the region within the far right part of the genome—there, some stop-gain mutations accumulated up to 2-digit percentages. The remaining mutations were all found within open reading frames (ORFs) encoding non-essential virus proteins, i.e., ORFs 6, 7b, and 8. The most frequently found mutation was 27858, introducing a stop codon in ORF7b at Q35, and 27945, within the open reading frame ORF8, there replacing the codon for Q18 with a stop codon. Of note, we observed highly frequent stop-gain mutations of ORF8 only in one of the virus pools selected with the nanobody, but not in the other nanobody-selected pool. Moreover, much of the immunomodulation by ORFs 6, 7b, and 8 can only be observed in an infected organism, not in a culture setting. Thus, there is no evidence that the ORF8 mutation was specifically selected by the nanobody.

To gain further insight into the composition of virus mutations introduced by NHC treatment and/or passaging, we determined stop-gain mutations, i.e., nonsense mutations that prevent the full-length synthesis of the protein corresponding to a particular open reading frame (ORF). Immediately after treatment with NHC, stop-gain mutations were distributed quite evenly across the virus genome. With further passaging, however, the number of such mutations decreased, whereas the few “surviving” stop-gain mutations increased in frequency (Figure 3). When analyzing the location of these maintained mutations in the genome, we only found them in ORFs 6, 7b, and 8, which are non-essential for coronavirus replication.^38^ The function of such ORFs consists of immunomodulation. We propose that such functions are not contributing to virus fitness in the context of a Vero E6 cell culture system, especially since the interferon system is partially deleted there.^39^ Thus, it is conceivable that only stop-gain mutations compatible with virus fitness were preserved during virus passaging, whereas all other stop-gain mutations were counterselected and lost.

### Distinct mutations are repeatedly selected when passaging the NHC-incorporated virus pool in the presence or absence of a neutralizing nanobody

**Figure 4:**
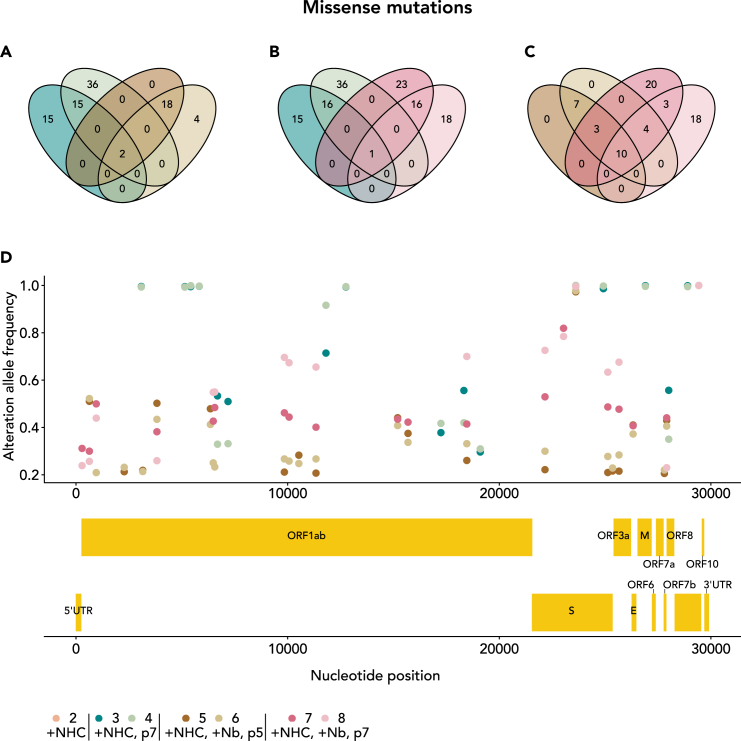
Repeated selection of distinct mutations, depending on the presence of nanobody Re5D06 (A–C) Venn diagrams to indicate the number of missense mutations found with high frequency (>0.2) in single or several virus pools. Comparison of virus pools obtained after serial passaging in the absence (#3, #4) or presence (#5, #6, #7, #8) of nanobody Re5D06 for two (A) or four (B) passages. Here, only a few missense mutations were found common to all such pools, but many mutations are common to the pairs of pools that were passaged in parallel using the same conditions. Comparison of virus pools obtained after passaging in the presence of the nanobody twice (#5, #6) or four times (#7, #8) (C). Note the high number of mutations common to all four pools. Similar results were obtained when plotting all detectable mutations in Venn diagrams, as shown in Figure S3A. (D) High-frequency (>0.2) missense mutations common to at least one pair of equally treated samples. Note that some mutations accumulated all across the genome when passaging the virus pool in the absence of nanobody (#3, #4). In contrast, passaging the viruses four times while adding the nanobody (#7, #8) led to a different spectrum of high frequency-mutations, with a cluster of such mutations found in the coding region of the spike protein. For orientation, a scaled depiction of the virus genome, including all known open reading frames, is displayed below the diagram.

We next asked whether different conditions, i.e., presence or absence of a neutralizing nanobody, lead to the accumulation of distinct sets of virus mutants. We compared lower and higher passages in the presence of nanobody with two experiments carried out in parallel for each condition. Comparing the four virus pools that were grown in the presence of the nanobody, we found a high degree of overlap between the identified mutations. On the other hand, when comparing the virus populations obtained with and without nanobody, we found far less overlap of mutations. This was true when restricting our analysis to missense mutations with a high frequency of >20% (Figures 4A–4C) but also when comparing all mutations regardless of frequency or codon (Figures S3A–S3C). Thus, the nanobody specifically and repeatedly gave rise to a similar spectrum of mutations.

Next, we compared the predominant mutations obtained in the presence or absence of the nanobody against the spike protein. Here, we focused our analysis on mutations that were selected with high frequency under one or several conditions. Mutations were often silent or without obvious functional relevance for the protein, e.g., displaying conserved charge or hydrophobicity (Figure S3D; Table S2). We filtered the mutations to select only missense mutations (Figure 4D). In the absence of nanobody, these high-frequency missense mutations occurred with roughly even distribution across the virus genome. In contrast, when passaged in the presence of nanobody against the spike protein, missense mutations were more clustered, mostly at the spike coding region. This finding suggests that the incubation with a nanobody gave preference to the selection of a virus pool with mutations in the spike protein, perhaps adapting to bypass the neutralization by the nanobody.

### Distinct mutations of the spike protein are selected by passaging the virus pool depending on the addition of anti-spike nanobody

**Figure 5:**
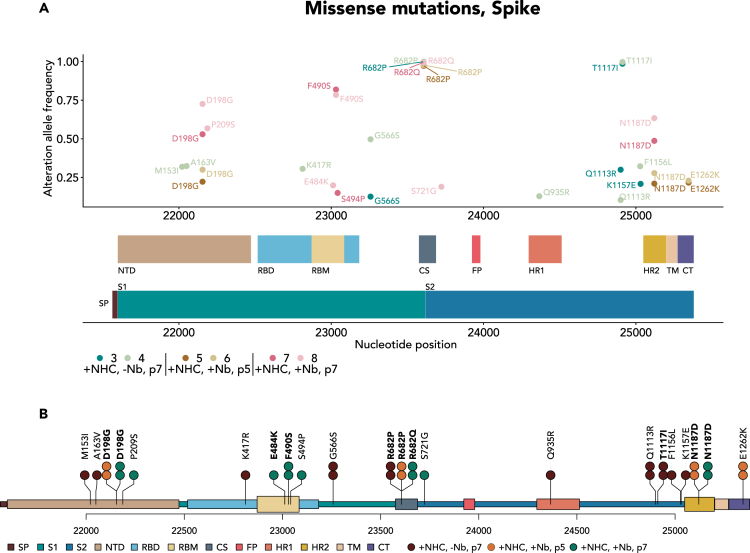
Characteristic spike mutations that explain gain of fitness, obtained by passaging the NHC-mutated pools of virus (A) As in Figure 4D, missense mutations found at a frequency of >0.2 in at least one of the virus pools are displayed, but this time confined to the region that encodes the virus spike protein. The resulting changes of the encoded amino acids are indicated. The domain structure of the spike protein is depicted underneath with the following domains: NTD, N-terminal domain; RBD, receptor-binding domain; RBM, receptor-binding motif; CS, cleavage site; FP, fusion peptide; HR1/2, heptad-repeat regions 1 and 2; TM, transmembrane domain; CT, carboxyterminal domain; SP, signaling peptide; S1 and S2, regions located N- and C-terminally from the Cleavage site. (B) The following mutations are indicated by lollipop symbols above the domain structure. ***R682P/Q***: These mutations were found in all virus pools that were passaged in Vero E6 cells, regardless of nanobody addition. The mutation eliminates a Furin cleavage site but allows for cathepsin cleavage of the spike protein into the S1 and the S2 domains and was previously found to promote virus propagation in Vero E6 cells.^41^ ***T1117I***: This mutation was only found in virus pools that were ***not*** under selective pressure with the nanobody. It is known from SARS-CoV-2 strains prevalent in Costa Rica, but its functional implications are unknown.^44^ Thus, the threonine residue at this position might confer some resistance in the presence of nanobody, whereas the isoleucine residue might increase neutralization. This could have many reasons, including the varying numbers of properly folded spike proteins within a virus particle. The following mutations were only selected when passaging the virus pool in the presence of nanobody Re5D06. ***F490S***: This mutation eliminates a phenylalanine residue that forms multiple interactions with the nanobody Re5D06, as revealed by the structure of the nanobody-RBD-complex^34^ (Figure 7) as well as AI-based structure predictions (Figure S6A). ***E484K***: This mutation removes a glutamate residue which also displays several interactions with nanobody Re5D06 (Figure 7), also suggested by structure prediction (Figure S6B). ***D198G***: The aspartate residue within the non-mutated spike protein was reported to form a salt bridge with the residue K462 of the RBD, presumably holding the RBD in the “down” conformation within the spike trimer.^45^ The mutation D198G might thus enhance the switch to the “up” conformation, perhaps facilitating virus entry in the presence of limiting amounts of nanobody. ***N1187D***: This mutation resides within the heptad repeat region 2 (HR2). It was found both at passage 5 and passage 7 in the presence of nanobody. It might modulate transient contacts with the HR1 after fusion of virus and cell membranes. HR1 and HR2 assume alpha helix conformations, and a bundle of six such helices is formed after fusion.^46^

Analyzing the accumulated mutations within the spike protein strongly suggested increased virus fitness, in particular concerning its replication despite the presence of an initially neutralizing nanobody. Firstly, however, passaging NHC-treated viruses under any condition led to the accumulation of the mutation p.R682P/Q (Figure 5A). This mutation deletes a Furin cleavage site (consensus: 679 RRAR 682, with cleavage occurring after p.R682) used for processing the virus spike protein between the domains S1 and S2.^40^ It was reported, however, that the loss of this site facilitates the propagation of SARS-CoV-2 in Vero E6 cells due to increased cleavage by cathepsins at the mutated S1/S2 site.^41^ Thus, the frequent occurrence of the mutation p.R682P/Q under all tested conditions strongly suggests gain of fitness. Unlike other SARS-CoV-2-susceptible cells, Vero E6 cells take up the virus through an endocytotic mechanism that does not depend on furin cleavage of the spike protein. Thus, the absence of the cleavage site provides the virus with a selective advantage, but only in Vero cells and not in other cell lines or experimental animals, as described previously.^2^^,^^42^^,^^43^

Most strikingly, however, selecting the virus pool with the nanobody led to the accumulation of the mutation p.F490S at a frequency of about 80% after prolonged passaging. Of note, p.E484K was also found under such conditions. F490 as well as E484 are both located within the receptor binding domain (RBD) of the spike protein, and they are also at the center of the interaction between the spike and nanobody Re5D06. We thus analyzed the structural consequences of these mutations in more detail, as described in the following.

Other accumulating mutations of the spike protein have less obvious explanations (Figure 5B). For instance, passaging the virus without nanobodies also led to the accumulation of the mutation T1117I (within the S2 domain but outside the heptad repeat regions or transmembrane domain), a mutation frequently found in variants isolated in Costa Rica^44^ without known functional implications.

The mutation p.D198G was also found with increased frequency in the nanobody-selected virus pools. The residue D198 within the N-terminal domain (NTD) of the spike was reported to form a salt bridge contact with residue K462 of the RBD in the “down” conformation.^45^ Changing this residue to a glycine might thus promote the “up” conformation of the spike. It is tempting to speculate that this will increase the chance of the RBD to contact the receptor protein ACE2 despite the presence of the neutralizing nanobody.

Moreover, growing the virus in the presence of nanobody led to the accumulation of the mutation p.N1187D within the Heptad Region 2 of the S2 domain. The heptad regions are essential for fusion of viral and cellular membranes. HR1 and HR2 undergo dramatic changes in conformation during this process, interacting with the membranes and forming a bundle of six alphahelices.^46^ The association of HR1 and HR2 might also represent a suitable target for peptide drugs to prevent membrane fusion upon virus entry.^47^ p.N1187 is right at the core of the fusion complex.^48^ Changing a neutral asparagine to a negatively charged aspartate residue within the alpha helix of HR2 might affect the stability of the bundle formed by the four HR1 and the two HR2 helices and affect the fusion process. p.N1187D is a characteristic mutation of the French virus variant B.1.616,^49^ indicating that this mutation is also viable in patients.

### NHC treatment enables earlier selection of nanobody-resistant virus mutants, compared to non-treated virus

**Figure 6:**
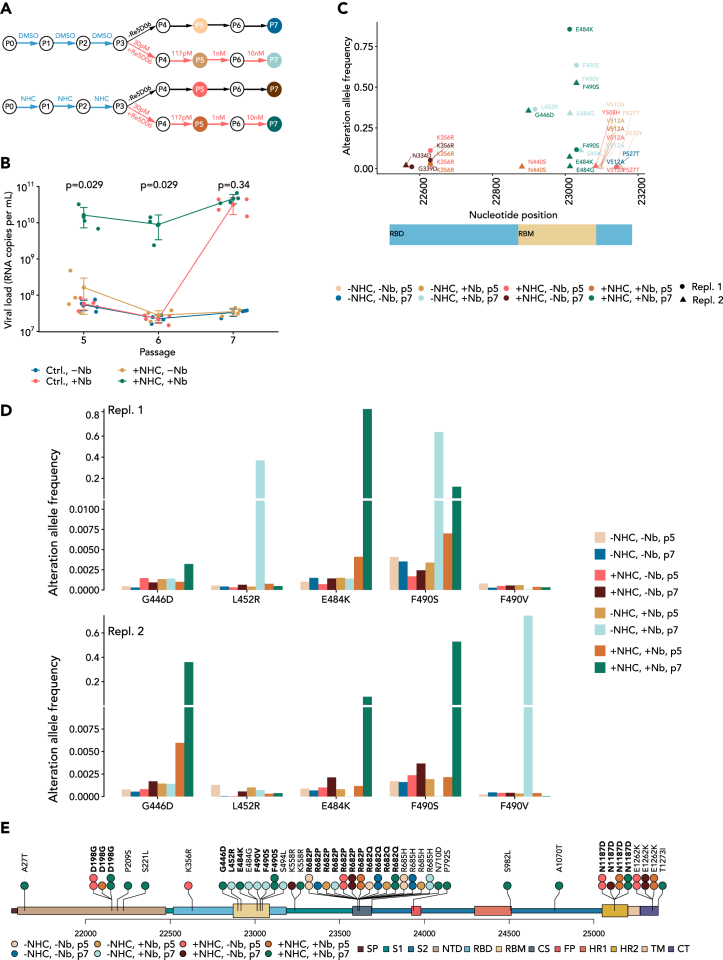
Direct comparison of the accumulation of nanobody-resistant virus mutants from virus pools that had been treated with NHC or were left untreated (A) Virus propagation. In a second selection experiment, the virus pools obtained from NHC-treated (Figure 1A) or DMSO-treated cells were passaged four times in the presence or absence of increasing amounts of the neutralizing nanobody, Re5D06 (n = 2). (B) Virus load in the presence of Re5D06. The virus-containing supernatants of higher passages (A) were used to infect a fresh monolayer of Vero E6 cells in the presence or absence of the nanobody at high concentration, i.e., 10 nM, to determine the degree of accumulated nanobody resistance at each passage. Data are represented as mean ± SD (n = 4). Notably, the observed resistance toward the nanobody was found at least two passages earlier in NHC-treated pools of virus, compared to control-treated virus pools. The significance of virus load difference between "Ctrl., +Nb" and "+NHC, +Nb" groups within each passage were estimated using Wilcoxon rank-sum test. Calculated p values from the test are shown above the dot plot. (C) Missense mutations found within the region encoding the RBD of the spike protein, in at least one of the virus pools, are displayed and the resulting changes of the encoded amino acids are indicated. In addition to the mutations ***F490S*** and ***E484K***, described in Figure 5 and found again here, NHC treatment and nanobody selection also led to the accumulation of ***G446D***, which impairs the interaction of the spike-RBD with the nanobody, as visualized in Figure 7. (D) Accumulation of missense mutations at virus passage 5 and 7. All eight virus pools were analyzed in two parallel experiments. The alteration allele frequencies of the most prominent mutations (i.e., G446D, L452R, E484K, F490S, and F490V) are shown. Note that the mutation G446D was enriched at passage 5 in replicate 2, then being the dominant mutation, whereas the F490S mutant became dominant at the later passage 7. (E) The predominant mutations depicted in (D) and in Tables S4 and S6 are indicated by lollipop symbols above the domain structure.

Finally, we compared NHC-treated and non-treated virus pools, side by side, as to the selection of nanobody-resistant mutants. The same virus preparation was passaged thrice in parallel, in the presence of NHC or the DMSO control. This was followed by incubations with nanobody Re5D06 (Figure 6A). The virus pools obtained at different passages were then tested as to their propagation in the presence of the nanobody. Remarkably, near-full resistance against the nanobody was found already two rounds after the nanobody was added (passage 5 in total) in the case of NHC treatment. In contrast, a non-NHC-treated virus pool took four passages with nanobody (passage 7 in total) to achieve resistance (Figures 6B and S4A). We then analyzed the accumulation of mutations by deep sequencing of the virus pools (Tables S4 and S5) and found the mutations p.F490S/V and p.E484K, as before (Figure 5), within the RBD of the spike protein (Figures 6C and S4B). Of note, however, one of the selections led to the mutation p.G446D, arguing that parallel *in vitro* evolution assays can lead to different solutions to achieve nanobody resistance. When the virus had not been exposed to NHC, selection with nanobody eventually led to resistance, too, albeit at later passages. These resistant virus pools displayed the mutations p.F490S/V and p.E484G, similar to the NHC-treated samples, but they also led to the accumulation of the mutation p.L542R. As outlined in the following, all these mutated residues are located at the interface of the spike protein and the nanobody.

The indicated mutations were present with high frequencies at passage 7 (Figure 6C). We then depicted their emergence at the earlier passage 5, comparing all eight virus pools (with and without NHC, with and without nanobody, passages 5 and 7; Figure 6D; Table S6). Interestingly, the selection—despite reaching resistance with similar kinetics—had followed two different pathways in two parallel experiments, each affecting the RBD (Figure 6E). In the first experiment, NHC facilitated the accumulation of the mutations p.E484K and (at lower frequency) p.F490S, the latter being the predominant mutation in the previous selections (Figure 5). NHC treatment allowed the accumulation of these mutations at low but detectable frequencies even at passage 5. Non-NHC-treated virus eventually displayed the p.F490S mutation at passage 7, but without detectable enrichment at passage 5 compared to virus pools that had not been confronted with nanobody. The second experiment led to the accumulation of p.G446D as well as p.F490S mutations (passage 7, NHC-treated virus). Interestingly, only the mutation p.G446D had been enriched at the earlier passage 5 in this experiment, as if this mutant was the dominant one at the earlier passage but was then partially outcompeted by the p.F490S mutant two passages later. It should be noted that each passage is likely to comprise several virus replication cycles, which might also explain why a relatively small proportion of virus mutants within a pool would nonetheless lead to a high amount of virus RNA copies in the test experiment (Figure 6B). Taken together, the direct comparison of NHC-exposed and non-exposed virus shows that NHC treatment considerably accelerates the emergence of antibody-resistant virus.

### The predominant nanobody-selected mutations p.G446D, p.L452R, p.E484K, and p.F490S of the spike protein are at the structural center of the spike-nanobody interaction

In the presence of nanobody Re5D06, mutations of SARS-CoV-2 were strongly selected that replaced the residues F490, E484, L452, and G446 of the spike protein. Indeed, the selected virus population was almost completely resistant to Re5D06, with no detectable reduction of virus propagation in the presence of this nanobody (Figures 1B and 1C). We therefore investigated whether these substitutions might compromise the interaction of the spike protein and its RBD with the nanobody while preserving its interaction with the virus receptor ACE2.

**Figure 7:**
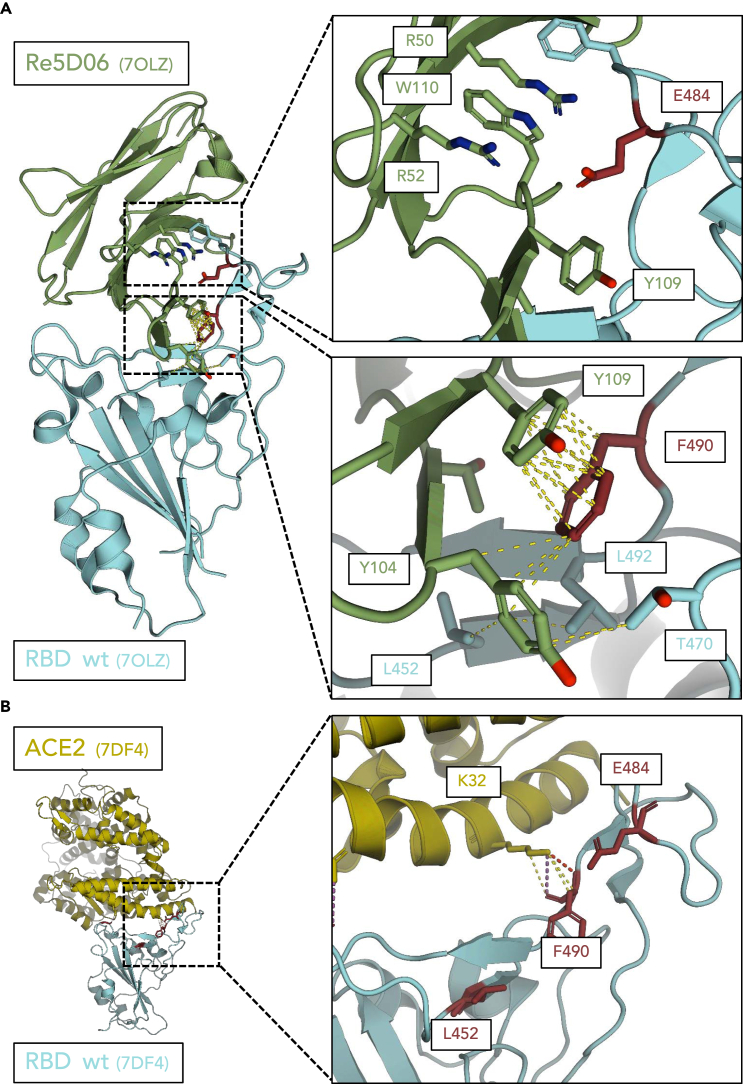
Structure analysis suggests that the interaction between spike and nanobody, but not between spike and ACE2, is compromised by the mutations L452R, E484K, and F490S (A) Interactions of the residues G446, L452, E484, and F490 of the spike protein with the nanobody Re5D06, based on the previously described structure of the complex.^34^ Hydrophobic interactions <4 Å are illustrated by yellow dashed lines. Electrostatic clashes are shown in red. The diminished affinity of the nanobody-RBD complex caused by the mutations L452R, E484K, and F490S can be explained as indicated below. In summary, the selected mutations are located at the site where the nanobody acts. The impact of the mutations on the interaction with the nanobody was also analyzed by AlphaFold predictions, as outlined in Figures S5 and S6. ***E484***—polar contacts between the nanobody residues R50, R52, and W110 and spike E484. The mutation spike E484K causes an electrostatic and steric clash with the nanobody at Y109 and W110. ***F490***—pi-stacking between nanobody Y110 and spike F490, lost with the spike F490S mutation. A hydrophobic cluster is formed between F490, T470, and L492of the RBD, as well as Y104, Y109, and T102 of the nanobody. This allows pre-orientation of F490 for interaction with the nanobody. ***L452***—The mutation L452R conceivably causes a steric clash with loop structure at nanobody K103 and Y104. (B) Role of the residues L452, E484, and F490 within the spike protein in the interaction between spike and ACE2, based on the previously reported structure of spike in a complex with ACE2.^52^ No direct interactions were observed between L452 and E484 and the ACE2 receptor. In the case of F490, interactions are only seen between the nanobody and the backbone of the spike. Sidechain rotamers are each oriented away from the interface, making it implausible that a mutation at these sites would grossly affect the affinity between the spike and ACE2.

We have previously reported the structure of the RBD in a complex with nanobody Re5D06^24^. As shown in Figure 7A, the mutated residues F490, E484, and L452 are in direct contact with the nanobody, making it plausible that their mutation compromises the interaction of the two proteins. In particular, the residue F490 enables hydrophobic π-stacking^50^ with nanobody Y109. The hydrophobic cluster arrangement pre-orients F490, allowing pre-orientation and entropic stabilization of the interaction with the nanobody. Interestingly, mutations of F490 were also found to provide resistance against different nanobodies against the spike.^51^ Polar interactions occur between RBD-E484 and nanobody R50, R52, and W110, conceivably stabilized by adjacent hydrophobic residues. Both L452 and F490 display hydrophobic contacts with nanobody Y104. The backbone of the RBD at G446 may support nanobody binding by greater backbone flexibility due to the lack of a beta carboatom. Furthermore, replacing G446 with an aspartate (D) residue may induce a spatial clash and/or electrostatic repulsion with the residue D30 of the nanobody, explaining why this mutation induces resistance.

The indicated mutations were selected during virus propagation in the presence of nanobody Re5D06. This not only raised the hypothesis that the mutations will disrupt the binding of the nanobody. It also suggested that the mutations should not counteract the binding of the virus to its receptor ACE2 through the spike RBD. The complex of the wild-type RBD with the ACE2 was previously found by cryo-electron microscopy.^52^ This experimentally determined structure did not reveal interactions between the residues E484 and L452 with the ACE2 (Figure 7B). In the case of F490, the lysine residue K32 of ACE2 only binds to the peptide backbone of the RBD at position 490 and does not interact with the phenylalanine ring. This at least suggests that the p.F490S mutation does not affect the binding to the ACE2 receptor.

Next, we sought to interrogate the role of these mutations in the interaction between the RBD and the nanobody in more detail. We used the program AlphaFold, which was established to predict the folding of proteins into tertiary structures with high accuracy.^53^ This program was recently further developed to predict the structures of protein complexes with more than one member.^54^ Using the program AlphaFold 2 Multimer, we modeled the interaction of Re5D06 and the RBD. Without mutations, the complex of the two proteins strongly overlapped with the structure that we had previously reported based on X-ray crystallography^34^ (Figure S5A).

We then subjected the same proteins for modeling, but with the mutations p.G446D, p.L452R, p.E484K, and p.F490S within the RBD. p.G446D did not grossly affect the predicted structure (Figure S5B). However, it should be noted that, despite strong overall accuracy, the AlphaFold prediction differed from the experimentally determined structure around G446 (Figure S7A) and may thus be unable to identify the consequences of a mutation at this residue. In the case of the three latter mutants, AlphaFold predicted a fundamental structural change of the complex, with a much smaller interaction interface^34^ with the nanobody (Figures S5B and S5C; Figure S6). This further adds plausibility to the concept that these mutations largely disrupt the proper interaction of the nanobody with the RBD.

To further investigate the impact of the most commonly found mutation p.F490S on the interaction between the RBD and ACE2, we modeled the complex of both proteins by AlphaFold, with or without the mutation F490S. The predicted structures of the complex did not grossly deviate from each other regardless of the mutation, and they were in agreement with the experimental structure (Figures S8 and S7B; Table S3). AlphaFold modeling further revealed that the substitution of p.F490 by S was entirely compatible with the molecular interaction of ACE2-K32 with the RBD (Figure 7B), thus strongly suggesting that the substitution within the RBD does not alter the binding of the spike to its ACE2 receptor.

These results should be taken within the limits of a structure prediction algorithm. The use of AlphaFold to model the effects of a single mutation on structures still awaits further evaluation. Limitations when analyzing single proteins in this way were reported,^55^ but the impact of mutations on inter-protein interactions,^56^ and in particular on the interactions of disease-associated protein mutants,^57^ was successfully evaluated using AlphaFold Multimer, adding validity to our approach.

Taken together, direct inspection of the RBD-nanobody structure as well as AlphaFold modeling are each compatible with the following concept: The substitutions p.G446D, p.L452R, p.E484K, and p.F490S allow the RBD to maintain its overall structure, preclude nanobody binding, and preserve ACE2 binding. In agreement, the mutations are also found in naturally occurring variants of SARS-CoV-2, i.e., p.F490S in variant Lambda^58^ and Omicron-XBB, p.L452R in B.1.427/429 and B.1.617,^59^ and p.E484K in B.1.351 and P.1.^60^ Similar to the nanobody, the mutation p.F490S also diminishes the binding of the therapeutic antibody Bamlanivimab to the spike.^61^ These properties are exactly what is required to allow virus propagation despite the presence of the otherwise neutralizing nanobody. Thus, NHC had induced mutations that were subsequently selected and allowed the virus to adapt and gain fitness to survive and spread in a hostile environment.

## Discussion

Our findings not only provide direct evidence that NHC, the active compound of Molnupiravir, is capable of inducing mutations in SARS-CoV-2 in an *in vitro* system. Even more remarkably, the virus pool obtained in a single flask of NHC-treated cells proved sufficient for the rapid development of cell-adapted and antibody-resistant virus mutants, and NHC considerably accelerated the development of resistance.

Overall, these findings strongly suggest that NHC treatment can give rise to virus mutants with increased fitness. However, our cell culture system is experimental in nature, and it is an open question whether this can be transferred to the situation of a Molnupiravir-treated COVID-19 patient. There can be little doubt that Molnupiravir will also give rise to virus mutants when used in the clinics. Indeed, recent reports provide evidence that such mutations are actually seen in patients.^62^^,^^63^^,^^64^^,^^65^ However, is there a substantial risk of treatment-induced gain-of-function SARS-CoV-2 mutants in patients? One could argue that a virus always acquires mutations as it spreads, just at a slower pace than upon treatment with Molnupiravir. However, the relatively late occurrence and pandemic spread of dominant SARS-CoV-2 mutants—namely, Delta and Omicron—suggests that the initial strain, even after infecting millions of individuals, remained comparatively stable. This argues that even one of the most pandemic coronaviruses in the world’s history does not rapidly fill the available sequence space to select the most infectious variants. Thus, it is difficult to fully exclude that using Molnupiravir in the clinics, despite all its benefits, might occasionally give rise to new virus mutants with increased capabilities in pathogenesis and transmission.

One concern would be that such virus mutants might quickly escape not only the immune response within an individual patient but also the antibody repertoire raised in a vaccinated population. In this context, it seems particularly worrisome that a small NHC-treated culture sample was capable of rapidly evolving resistance to one of the most potently neutralizing antibody structures reported so far, i.e., nanobody Re5D06.^34^ Even without providing this system with any information on the structural features of the nanobody-spike complex, it still selected spike mutations precisely at the center of the interface, in each case largely abolishing the interaction by exchanging just one amino acid residue. Thus, at least in principle, Molnupiravir has a strong potential of accelerating the emergence of viral immune escape variants.

Besides immune escape, virus variants may also be selected to accelerate transmission between individuals. This can be achieved by increased stability of the infectious particles, by shifted cell tropism but perhaps most conceivably by enhanced efficiency of the pre-established viral entry mechanism. At least in the experimental system presented here, the most impressive mutagenesis was found within the spike protein. Not only the RBD as such was subject to nanobody-escape mutations. Mutations at the cleavage site of the spike were also found particularly often. Hence, mutations were selected to affect the virus-receptor interactions and the fusion of membranes, and this represents an important opportunity for coronaviruses to vary infectivity and cell tropism. Such mechanisms appear to gain momentum by a drug that mutates the virus genome. Thus, there is a concern that transmissible mutants could occur if Molnupiravir is used inappropriately, especially when treating patients with suboptimal doses, or for insufficient durations, and without isolating them. However, if used with appropriate care, the continued, controlled use of Molnupiravir might still be beneficial.

A third concern is that Molnupiravir may promote the resistance of viruses against the drug itself or against additional therapeutics that were applied together with it. Since Molnupiravir does not work through direct inhibition of viral enzymes, Molnupiravir-resistant virus mutants may be unlikely to occur. And indeed, even prolonged NHC treatment did not lead to resistance formation in other coronaviruses.^66^ However, resistant virus mutants are more conceivable when combining Molnupiravir with therapeutic antibodies or with inhibitors of viral proteases, as contained in the recently approved drug Paxlovid.^67^ Indeed, mutations at single residues can render SARS-CoV-2 more resistant against Paxlovid.^68^ These considerations argue against the use of combinations of Molnupiravir and additional drugs in therapy, despite their efficacy in preclinical model systems.^69^

Beside the mutagenesis in virus genomes, another concern of using Molnupiravir consists in the possible risk of mutagenesis in the DNA of patient cells. Such mutations, if they occur, might give rise to cancer. In pregnant women, they might also cause damage to the embryo. Currently, there is limited data to support such a scenario. Prolonged treatment of cultured cells with NHC for several weeks gave rise to low numbers of *HPRT1*-deficient mutants,^23^ leaving doubts whether such a degree of mutagenesis might be relevant in people receiving Molnupiravir only for a few days. DNA mutagenesis by NHC presumably depends on the reduction of NHC (in its diphosphorylated form) at the 2′ position of its ribose to become a deoxyribonucleotide. Only then will NHC metabolites be incorporated into the DNA. The degree to which this happens remains to be determined.

Why did we observe the accumulation of different mutations in parallel experiments with seemingly identical conditions? As in the real world, identical conditions can lead to the evolution of different variants of an organism, since stochastic processes play an important role and since several genetic solutions can often be found to meet a phenotypic challenge.^70^ Since less mutations are occurring in the starting material when NHC is absent, there is an even higher chance that sometimes even suboptimal “solutions” (i.e., mutations) are selected by the challenge (i.e., the nanobody). Thus, it is not surprising that genetically different results occurred in the two replicas. For instance, when selecting with nanobody from non-NHC-treated virus pools, both selections led to a mutation of F490 at the highest frequency. However, one experiment selected the mutation F490S, the other one selected F490V (Figure 6D). Still, both mutations remove the most critical amino acid residue within the spike to bind the nanobody.

Molnupiravir is not the only drug that develops its antiviral activity through mutations in the virus genome.^26^ Another prominent example is Ribavirin, since its mechanism of action is also, at least partially, based on mutagenesis of virus RNA.^71^ Ribavirin has been used for decades in treating Hepatitis C, and at least up to now, no extensive occurrence of virus mutants was reported in these patients. However, the selection of viruses with enhanced fitness might depend on a number of parameters specific for each virus. They include the average tolerance of viral proteins for mutations with regard to their functions; the immunogenicity of virus mutants; and the rate of virus replication, reflecting opportunities of incorporating mutations. The same holds true when using Molnupiravir against infections with other viruses in the future. In preclinical models, for instance, Molnupiravir or NHC were already found active against influenza virus^72^^,^^73^ or Ebola virus.^74^

Further studies are required to assess the occurrence of mutant SARS-CoV-2 in Molnupiravir-treated animals and patients. However, even the available data are raising concerns regarding the use of Molnupiravir outside a controlled clinical setting. We propose that treatment with Molnupiravir should be limited to patients at high risk of developing severe COVID-19. Treatment doses and duration should be chosen to eliminate the virus below detectability. And the patients, while still being infectious, should avoid social contacts to limit the possible spread of virus variants that were formed due to Molnupiravir-induced mutagenesis.

### Limitations of the study

The experiments shown here were all performed *in vitro*, i.e., in a cell culture system and using a single species of a spike-binding nanobody. This is different from the *in vivo* situation by several aspects: An infected body replicates the virus with different kinetics and in multiple cell types. These cells are likely to take up Molnupiravir and its metabolites with different efficacies. The immune response comprises a multitude of antibodies, flanked by T cells as well as the natural immune response. Finally, the virus spreads to other individuals through aerosols, not through incubation with tissue culture supernatant. In addition to these considerations, it should also be noted that we deliberately used a suboptimal dose of NHC in our study. Obviously, an NHC dose that eliminates all virus would not give rise to any mutants. Conceivably, keeping drug concentrations well above full efficiency levels would likely reduce the likelihood of raising escape mutants *in vivo*, too. Thus, the results of our study should be taken as what they are: a strongly simplified model system that reflects only single aspects of the infection of an organism. We show that Molnupiravir has the potential of selecting antibody-resistant virus. It remains to be seen whether this actually happens in a real-world setting, especially when patients are treated with Molnupiravir without fully suppressing virus propagation.

## STAR★Methods

### Key resources table

**Table:** 

REAGENT or RESOURCE	SOURCE	IDENTIFIER
**Antibodies**		

SARS-CoV-2 Nucleoprotein	Sino Biological	Cat# 40143-R019; RRID: AB_2827973
Alexa Flour 546 donkey anti-rabbit	Thermo Fisher Scientific	Cat# A10040; RRID: AB_2534016

**Bacterial and virus strains**		

SARS-CoV-2 ‘wildtype’, Göttingen/Germany	Isolated from patient (Stegmann et al.)^35^	N/A

**Chemicals, peptides, and recombinant proteins**		

β-D-N^4^-Hydroxycytidine (NHC/EIDD-1931)	Cayman Chemical	Cat# 9002958
DMSO	Applichem	Cat# A3672.0100
Nanobody Re5D06	Güttler et al.^34^	N/A
Lysis Binding buffer (from MagNA Pure LC Total Nucleic Acid Isolation Kit)	Roche	Cat# 03038505001
Trizol LS	Life Technologies	Cat# 10296028
Trichlormethan/Chloroform	Applichem	Cat# 3313.1
Isopropanol	Applichem	Cat# 6752.2
Ethanol	ChemSolute/Th.Geyer	Cat# 11647081/2246
Triton X-100	Applichem	Cat# A1388
4′,6-Diamidino-2- Phenylindole (DAPI)	Sigma	Cat# D9542-5MG

**Critical commercial assays**		

TruSeq RNA Library Preparation Kit v2, Set A (48 samples, 12 indexes)	Illumina	RS-122–2001
dsDNA 905 Reagent Kit	Advanced Bioanalytical	DNF-905

**Deposited data**		

ma-qp22j (RBDwt-Re5D06)	ModelArchive	ModelArchive: ma-qp22j
ma-0pnx1 (RBD p.G446D-Re5D06)	ModelArchive	ModelArchive: ma-0pnx1
ma-v4odj (RBD p.L452R-Re5D06)	ModelArchive	ModelArchive: ma-v4odj
ma-jgqs4 (RBD p.E484K-Re5D06)	ModelArchive	ModelArchive: ma-jgqs4
ma-r62mw (RBD p.F490S-Re5D06)	ModelArchive	ModelArchive: ma-r62mw
ma-lxnac (RBDwt-ACE2)	ModelArchive	ModelArchive: ma-lxnac
ma-eh742 (RBD p.F490S-ACE2)	ModelArchive	ModelArchive: ma-eh742
Sequencing data PRJEB65430 (ERP150548)	European Nucleotide Archive	European Nucleotide Archive: ERP150548

**Experimental models: Cell lines**		

*Monkey*: Vero E6 (Vero C1008)	ATCC	Cat# CRL-1586

**Oligonucleotides**		

Primer (probe), with 5′FAM, 3′BBQ ACA CTA GCC ATC CTT ACT GCG CTT CG	Eurofins Genomics	N/A
Primer (forward) ACA GGT ACG TTA ATA GTT AAT AGC GT	Eurofins Genomics	N/A
Primer (reverse) ATA TTG CAG CAG TAC GCA CAC A	Eurofins Genomics	N/A

**Software and algorithms**		

Prism (version 9.0.0)	GraphPad	N/A
BioRender	BioRender	N/A
FastQC (version v0.11.9)	Andrews^77^	
trim_galore (version 0.6.7)	https://www.bioinformatics.babraham.ac.uk/projects/trim_galore/	N/A
kraken2 (version 2.1.2)	Wood and Salzberg^78^	N/A
BWA-mem (version 0.7.17-r1188)	Li and Durbin^79^	N/A
samtools (version 1.15)	Li et al.^80^	N/A
iVar (version 1.3.1)	Grubaugh et al.^81^	N/A
FreeBayes (version v1.3.6)	Garrison and Marth^82^	N/A
vcfbreakmulti from the vcflib package (version 1.0.3)	Garrison et al.^83^	N/A
SnpEff (version 4.5covid19)	Cingolani et al.^84^	N/A
R 4.2.2	N/A	N/A
trackViewer R package	Ou and Zhu^85^	N/A
AlphaFold-Multimer version 2.1.1 and 2.2.0	N/A	N/A

### Resource availability

#### Lead contact

Further information and requests for resources and reagents should be directed to and will be fulfilled by the lead contact, Matthias Dobbelstein (mdobbel@uni-goettingen.de).

#### Materials availability

This study did not generate new unique reagents.

### Experimental model and study participant details

Vero E6 cells (Vero C1008) were obtained from the German Primate Research Center Göttingen. Cells were maintained in Dulbecco’s modified Eagle’s medium (DMEM with GlutaMAX™, Gibco) supplemented with 10% fetal bovine serum (FBS; Merck), 50 units/mL penicillin, 50 μg/mL streptomycin (Gibco), 2 μg/mL tetracycline (Sigma) and 10 μg/mL ciprofloxacin (Bayer) at 37°C in a humidified atmosphere with 5% CO_2_. Vero E6 cells were authenticated in 2021 by means of Cytochrome C Subunit I (COI) DNA Barcoding by the Deutsche Sammlung von Mikroorganismen und Zellkulturen GmbH (DSMZ). Furthermore, the cells were routinely tested to ensure they were negative for mycoplasma contamination, using the MycoAlert Assay Control Set (Lonza).

### Method details

#### Treatments and SARS-CoV-2 infection

250,000/750,000 cells were seeded into T25/T75 flasks, respectively, using medium containing 2% FBS, and incubated for 8 h at 37°C. Cells were treated with β-D-N^4^-hydroxycytidine (NHC/EIDD-1931, Cayman Chemical 9002958) at the concentrations indicated in the legends to Figures 1 and 6. After 24 h, cells were infected with SARS-CoV-2 and incubated for 48 h at 37°C. For the selection of nanobody-resistant mutants, increasing concentrations of Nanobody Re5D06^34^ were pre-mixed with SARS-CoV-2 and incubated for 1 h at 37°C. Afterwards, the nanobody-virus-mix was added to the cells for 48 h at 37°C. The SARS-CoV-2 strain used in these experiments was isolated from a patient sample taken in March 2020 in Göttingen, Germany.^35^

#### Quantitative RT-PCR for virus quantification

For RNA isolation, 100 μL of the SARS-CoV-2-containing cell culture supernatant was mixed (1:1 ratio) with the Lysis Binding Buffer from the MagNA Pure LC Total Nucleic Acid Isolation Kit (Roche) to inactivate the virus. The viral RNA was isolated using Trizol LS, chloroform, and isopropanol. After washing the RNA pellet with ethanol, the isolated RNA was re-suspended in nuclease-free water. Quantitative reverse transcription and polymerase chain reaction (RT-PCR) was performed involving a TaqMan probe,^75^ to quantify virus RNA yield. The following oligonucleotides were used for RT-PCR, which amplify a genomic region corresponding to the envelope protein gene (26,141–26,253).^75^

**Table:** 

Primer	Sequence	Modification
P (probe)	ACA CTA GCC ATC CTT ACT GCG CTT CG	5′FAM, 3′BBQ
F (forward)	ACA GGT ACG TTA ATA GTT AAT AGC GT	
R (reverse)	ATA TTG CAG CAG TAC GCA CAC A	

#### Immunofluorescence microscopy for TCID50 determination

To determine the Median Tissue Culture Infectious Dose (TCID_50_), the virus-containing supernatant was titrated (endpoint dilution assay). Vero E6 cells were seeded in 96-well plates and incubated with 10-fold dilutions (3–4 technical replicates) of virus for 48 h and then fixed with 4% formaldehyde in PBS for 1 h at room temperature. After permeabilization with 0.5% Triton X-100 in PBS for 30 min and blocking in 10% FBS/PBS for 10 min, a primary antibody was used to stain the SARS-CoV-2 Nucleoprotein (N; Sino Biological #40143-R019, 1:8000). The secondary Alexa Fluor 546-coupled donkey anti-rabbit IgG antibody (Invitrogen, 1:500, diluted in blocking solution) was added together with 4′,6-diamidino-2-phenylindole (DAPI) for 1.5 h at room temperature. Fluorescence signals were detected by automated microscopy using a Celígo® Imaging cytometer. The titer was determined according to Spearman and Kärber.^76^

#### Deep sequencing

DNA sequencing was performed as follows.^35^ RNA-seq libraries were prepared using the Illumina; TruSeq RNA Library Preparation Kit v2, Set A; 48 samples, 12 indexes, Cat. N°RS-122–2001. Adapter ligation efficiency was >94%, and we reduced the number of PCR cycles to 10. The size of final cDNA libraries was determined at ∼280 bp using the dsDNA 905 Reagent Kit (Advanced Bioanalytical). Libraries were pooled and sequenced on the Illumina HiSeq 4000 analyzer (PE; 1 × 2 × 150 bp; 80 Mio reads/sample).

#### Sequencing data processing and variant calling

FastQC (version v0.11.9)^77^ was first used to evaluate the read quality of raw FASTQ files. As a cleanup step, trim_galore (version 0.6.7, https://www.bioinformatics.babraham.ac.uk/projects/trim_galore/) was used to trim the adapters, followed by removing the contaminating human host reads by kraken2 (version 2.1.2).^78^ The cleaned sequencing reads were then aligned to the SARS-CoV-2 reference genome (NC 045512.2) using BWA-mem (version 0.7.17-r1188).^79^ The resulting BAM alignments were sorted and indexed by samtools (version 1.15).^80^ We then applied iVar (version 1.3.1)^81^ to trim the primer sequences from the aligned and sorted BAM files. FreeBayes (version v1.3.6)^82^ was used to perform variant calling, thresholded on minimum alternate allele fraction of 0.01. The yielded VCF files were splitted using vcfbreakmulti from the vcflib package (version 1.0.3).^83^ Variants were annotated using SnpEff (version 4.5covid19).^84^

#### Variant distribution visualization

SARS-CoV-2 genome annotation GCF_009858895.2_ASM985889v3_genomic.gff was obtained from https://www.ncbi.nlm.nih.gov/sars-cov-2/. The filtered alteration allele frequency along genome was plotted against the nucleotide positions using R 4.2.2. Mutation lolliplot was made using trackViewer R package.^85^

#### Structure predictions by AlphaFold

Predictions were carried out using AlphaFold-Multimer version 2.1.1 and 2.2.0. The Multiple Sequence Alignments (MSAs) used for the structure inference were built with the standard AlphaFold pipeline. Template modeling was enabled, but only templates that were released before 12–2019 were used. To model the RBD-ACE2 dimer, we also included more recent templates. As AlphaFold only uses single-chain templates and excludes templates with identical sequence, a simple replication of published structures is impossible.

Structures were inferred with eight MSA recycling iterations and all five different model parameter sets. After prediction, models were ranked by the pLDDT score for monomers and pTM score for dimers.

Predictions are available in ModelArchive (modelarchive.org) with the accession codes ma-qp22j (RBDwt-Re5D06), ma-0pnx1 (RBD p.G446D-Re5D06), ma-v4odj (RBD p.L452R-Re5D06), ma-jgqs4 (RBD p.E484K-Re5D06), ma-r62mw (RBD p.F490S-Re5D06), ma-lxnac (RBDwt-ACE2), and ma-eh742 (RBD p.F490S-ACE2). Model confidence predictions per residuum (pLDDT-score) are stored in the b-factors column of the .pdb file. PAE-value-plots for each structure are included in the ModelArchive entries.

All predictions were performed using the high-performance computer ‘Raven’, operated by the Max-Planck Computing & Data facility in Garching, Munich, Germany.

### Quantification and statistical analysis

Virus titration data are represented as mean ± SD (n = 4). The significance of virus load difference between "Ctrl., +Nb" and "+NHC, +Nb" groups within each passage were estimated using Wilcoxon rank-sum test. Statistical details of each experiment can be found in the corresponding figure legend.

## Data Availability

• All data reported in this paper will be shared by the lead contact upon request. • Predictions have been deposited in ModelArchive (Accession numbers: ma-qp22j, ma-0pnx1, ma-v4odj, ma-jgqs4, ma-r62mw, ma-lxnac, ma-eh742) and are publicly available. Sequencing data have been deposited in European Nucleotide Archive (RJEB65430 [ERP150548]). Accession numbers are listed in the key resources table. Any additional information required to reanalyze the data reported in this paper is available from the lead contact upon request. • This paper does not report original code. Any additional information required to reanalyze the data reported in this paper is available from the lead contact upon request.

## Supplemental information

Document S1. Figures S1–S8

Table S1. Concentrations of virus RNA and drugs, related to Figure 1 For all steps depicted in Figure 1A, labeled by letters, the number of virus RNA genomes used to inoculate the cells are indicated, as well as the amount of virus RNA obtained after infection (corresponding to virus yield). The table also indicates the numbers of cells used for every round of virus propagation, as well as the amount of NHC or nanobody added to the media.

Table S2. Single nucleotide variations within virus genomes, related to Figures 2, 3, 4, and 5 A comprehensive presentation of all sequencing results is provided, corresponding to Figures 2, 3, 4, and 5. Each mutation of the virus genome found in any of the samples above background is indicated, along with its frequency in each sample and corresponding amino acid changes in coding regions.

Table S3. Complex sequence variations within virus genomes, related to Figure 2 As in Table S2, the results of deep sequencing are provided, this time displaying complex variations such as deletions.

Table S4. Single nucleotide variations upon comparative treatment with or without NHC and/or nanobody, related to Figure 6 A comprehensive presentation of the sequencing results is provided, corresponding to Figure 6 and in analogy to Table S2.

Table S5. Complex sequence variations within virus genomes, related to Figure 6 As in Table S4, the results of deep sequencing are provided, this time displaying complex variations such as deletions.

Table S6. Key alterations within the RBD, related to Figure 6 Key alterations of the RBD, as displayed in Figure 6D, are presented even if present at low frequency (down to 0.1%).
