# Supplementary material for: N4-hydroxycytidine, the active compound of Molnupiravir, promotes SARS-CoV-2 mutagenesis and escape from a neutralizing nanobody
Source: iScience. 2023 Aug 30;26(10):107786. doi: 10.1016/j.isci.2023.107786 (PMC10507161; doi:10.1016/j.isci.2023.107786)

iScience, Volume *26*

Supplemental information

N4-hydroxycytidine, the active compound of Molnupiravir, promotes SARS-CoV-2 mutagenesis and escape from a neutralizing nanobody

Arne Zibat, Xiaoxiao Zhang, Antje Dickmanns, Kim M. Stegmann, Adrian W. Dobbelstein, Halima Alachram, Rebecca Soliwoda, Gabriela Salinas, Uwe Groß, Dirk Görlich, Maik Kschischo, Bernd Wollnik, and Matthias Dobbelstein

FIGURE S1: Acquired resistance towards nanobody Re5D06, as determined by $TCID_{50}$, Related to Figure 1C

The virus-containing supernatant of Figure 1B was titrated on 96-well-plates to determine the Median Tissue Culture Infectious Dose ($TCID_{50}$, n=4). Fluorescence signals were detected by automated microscopy. Note that the virus populations that had been obtained by passaging in the presence of the nanobody (#7, #8) were capable of replicating efficiently even when the nanobody was added. In contrast, the virus populations obtained at the same number of passages in plain media (#3, #4) were now still neutralized by the nanobody.

FIGURE S2: Allele frequencies of transitions and transversions, Related to Figure 2

From the samples described in Figure 1A, the frequencies of mutations are indicated in the context of specific base triplets. Note that transitions between G and A (green) as well as between T/U and C (purple) were far more frequent than transversions between purines and pyrimidines upon treatment with NHC. Similar mutations were becoming even more frequent upon passaging of the virus pools.

FIGURE S3: Location of all high-frequency mutations in the virus populations obtained after passaging with or without nanobody, Related to Figure 4

(A-C) Venn diagrams to indicate the number of all mutations found at >20% frequency in at least one virus pool. The diagrams were established in analogy to Figure 4, A-C, but comprising all mutations rather than just missense mutations.

(D) All high-frequency mutations (>20%) found in at least one sample, including silent, missense and stop-gain mutations, are displayed along the virus genome, in analogy to Figure 4D.

FIGURE S4: Infectivity of the virus and location of missense mutations encoding the spike protein, comparing NHC-treated and control-treated virus pools, Related to Figure 6

(A) The virus-containing supernatant of Figure 6B was titrated on 96-well-plates to determine the Median Tissue Culture Infectious Dose ($TCID_{50}$, n=4).

(B) Missense mutations found at >10% in at least one of the sample obtained after selecting NHC-treated and control-treated virus pools, as described in Figure 6A. The same data, but confined to the RBD, are presented in Figure 6C.

FIGURE S5: AlphaFold predictions add plausibility to the impairment of the spike-nanobody-interaction by the mutations G446D, L452R, Related to Figure 7

The structures of the heterodimers, consisting of nanobody Re5D06 (green) with the Receptor Binding Domain (RBD) of the SARS-CoV-2 spike protein in its wildtype (A) and its mutants (B-E) versions, were predicted by AlphaFold.

The key residue positions 446, 452, 484 and 490 are shown in red. Mutated residues are shown in yellow. Interactions with the nanobody are shown by dashed lines. Polar contacts <4Å are shown in magenta, and other contacts <4Å are colored with yellow dashed lines. Electrostatic clashes are shown in red. The middle panels represent enlarged portions of the left panels; structures on the right panel are colored by the pLDDT value, measuring the prediction confidence, according to the indicated color code.

(A) Wildtype RBD in complex with the nanobody. The predicted structure is very similar to the experimentally (X-ray) structure that was reported previously [24] and indicated in Fig. 7A. The overlaps between the prediction and the experiment are shown in Figure S7A. Note, however, that the prediction confidence (pLDDT value) is low near the loop structure between T28 and D30 of the nanobody, where the prediction is also deviating from the experimentally determined structure (cf. Figure 6A).

(B) RBD p.G446D in complex with the nanobody. Due to the local deviation of the AlphaFold prediction and the experimentally determined structure (cf. legend to A), the phenotype cannot be explained by AlphaFold in this case. In particular, the conceivable clash between nanobody-D32 and RBD-D446 cannot be seen in this model.

(C) RBD p.L452R in complex with the nanobody. This single point mutation is sufficient to disrupt the initially predicted structure of the complex. Instead, AlphaFold now predicts a distorted, implausible structure, with little interaction of the two proteins and a low confidence (pLDDT) at the interface. Moreover, the beta-sheet at nanobody-T102 and Y109 is no longer predicted. The lack of a predicted high-confidence complex structure strongly agrees with the observed loss in neutralization.

FIGURE S6: AlphaFold predictions add plausibility to the impairment of the spike-nanobody-interaction by the mutations E484K and F490S, Related to Figure 7

The color code was used as in Fig. S7.

(A) RBD p.F490S in complex with the nanobody. When the phenylalanine residue F490 is replaced by serine, AlphaFold no longer predicts a plausible interaction with the nanobody. Instead, most of the molecular interactions with these proteins are no longer predicted, leaving only a minor interface for interaction. S490 in the mutant RBD shows interaction with W110 of the nanobody, but the overall association between the mutant RBD and the nanobody is limited and differently oriented. This strongly agrees with the central role of spike-F490 in the formation of the complex, including its multiple interactions with Y109 and Y104 of the nanobody (panel A and Fig. 7A)

(B) RBD p.E484K in complex with the nanobody. Again, this substitution led to the prediction of an implausible complex with a distorted interface between the RBD and the nanobody, as compared to the wildtype-RBD (A). This is in agreement with the observed accumulation of this mutation in a pool of highly nanobody-resistant virus.

FIGURE S7: AlphaFold predictions in agreement with the experimentally determined structures, Related to Figure 7

(A) Superimposition of the RBD in complex with the nanobody Re5D06, comparing the AlphaFold prediction with the experimentally determined structure 7OLZ [24]. The root mean square deviation (RMSD) of the aligned pairs of the backbone C-alpha atoms, when superimposing the experimental RBD structure from 7OLZ with the AlphaFold-predicted structure, is 0.4. Comparing the nanobody structures in the same setting, the RMSD is 0.6. And the RMSD for the entire complex is 1.9.

(B) Superimposition of the RBD in complex with the ACE2 receptor, predicted by AlphaFold, and the experimental structure 7DF4 [40]. Here, the RMSDs were 1.1 for the RBD; 1.8 for the ACE2 structure; and 2.1 for the complex of both.

FIGURE S8: Preserved structure of the spike receptor binding domain (RBD) and ACE2 within their complexes, as predicted by AlphaFold, Related to Figure 7

Preserved interaction of the spike protein and ACE2, despite the substitution p.F490S. The substitution p.F490S does not affect the interactions of the RBD with its natural receptor ACE2. The lysine residue K31 on the ACE2 interacts with the spike protein at position F490; however, it only forms molecular interactions with the backbone of the peptide chain, not with the phenylalanine residue (upper panel). The replacement of F490 with a serine residue (lower panel) does not alter these interactions. Also, no major structural changes were observed when substituting F490 with an S residue. Wildtype and mutant RBD (cyan) are each predicted by AlphaFold (v. 2.1.1) to associate with ACE2 (yellow) in similar ways. Moreover, the predicted structure of this complex almost completely coincides with the experimentally determined structure PDB 7DF4 [40] as displayed in Figure S7B.

Figure S1, related to Figure 1

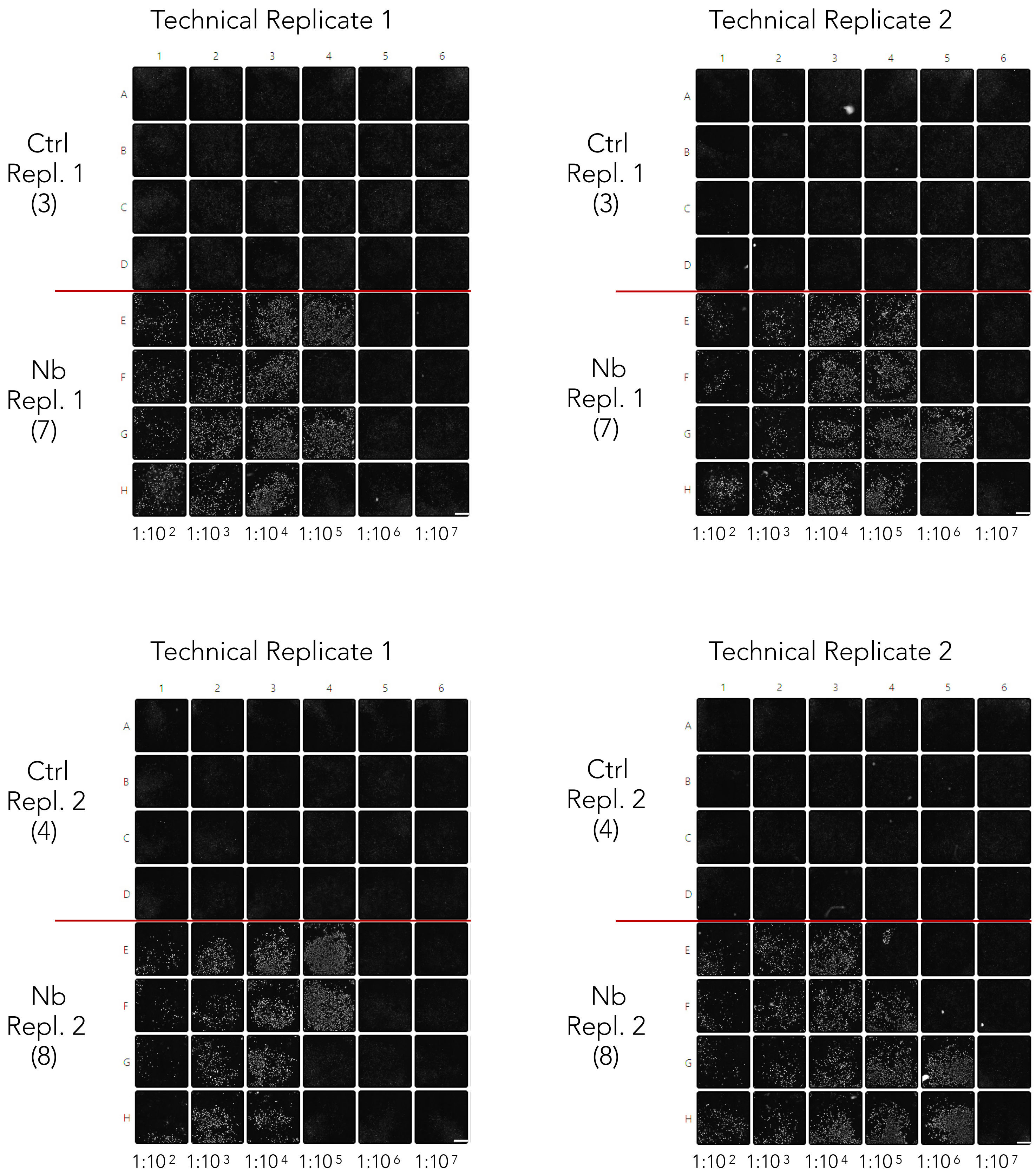

Figure S2, related to Figure 2

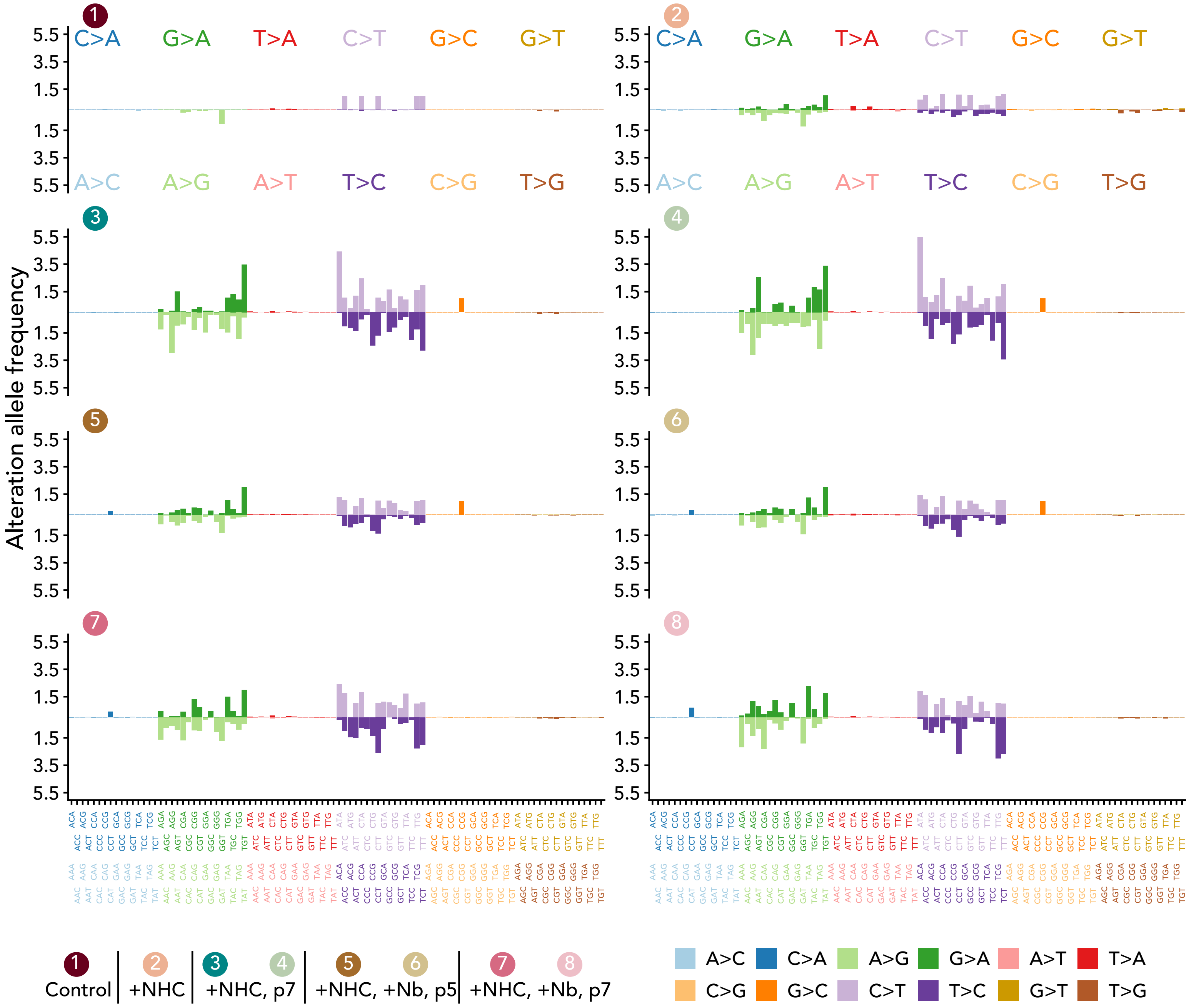

Figure S3, related to Figure 4

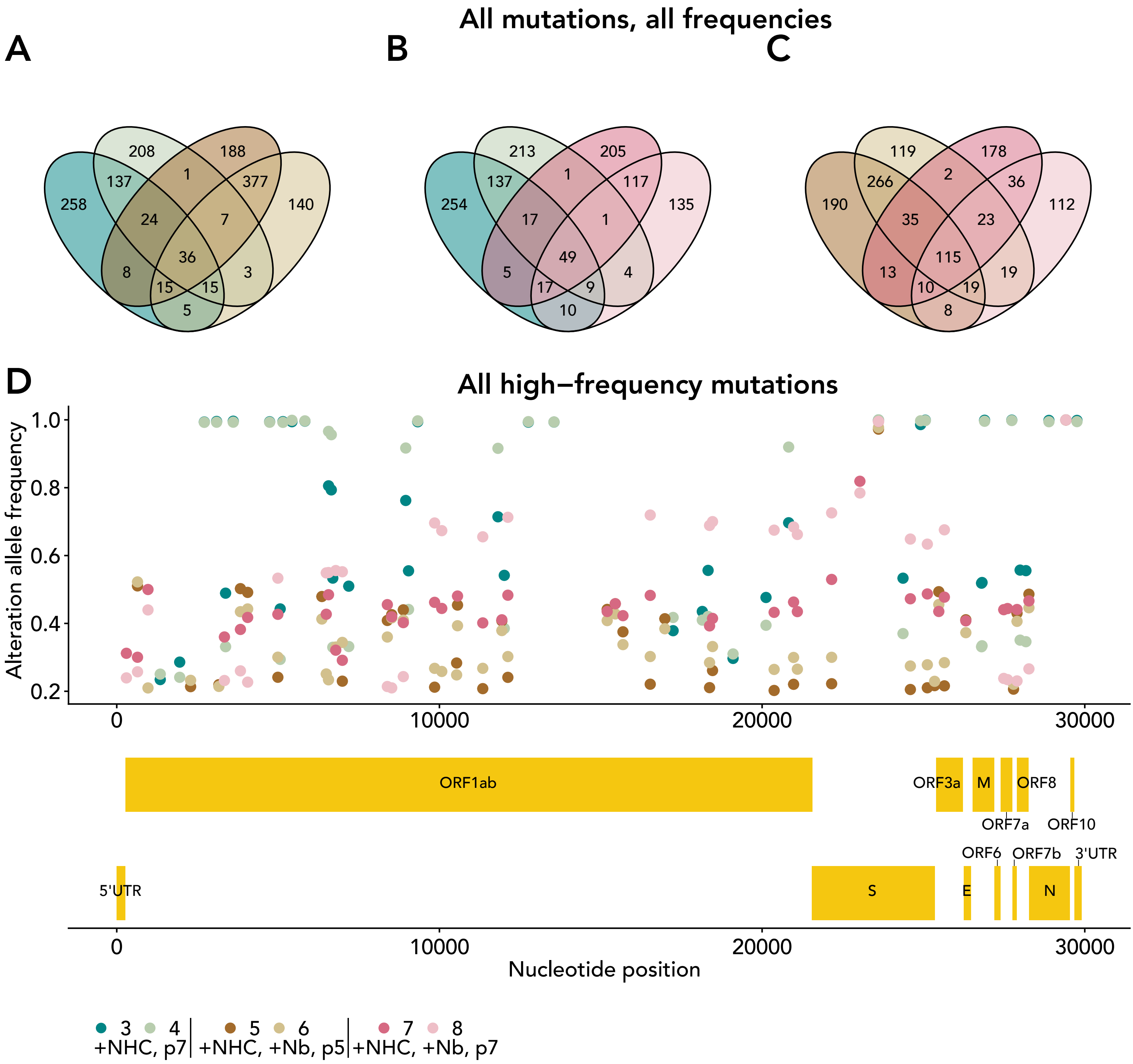

Figure S4, related to Figure 6

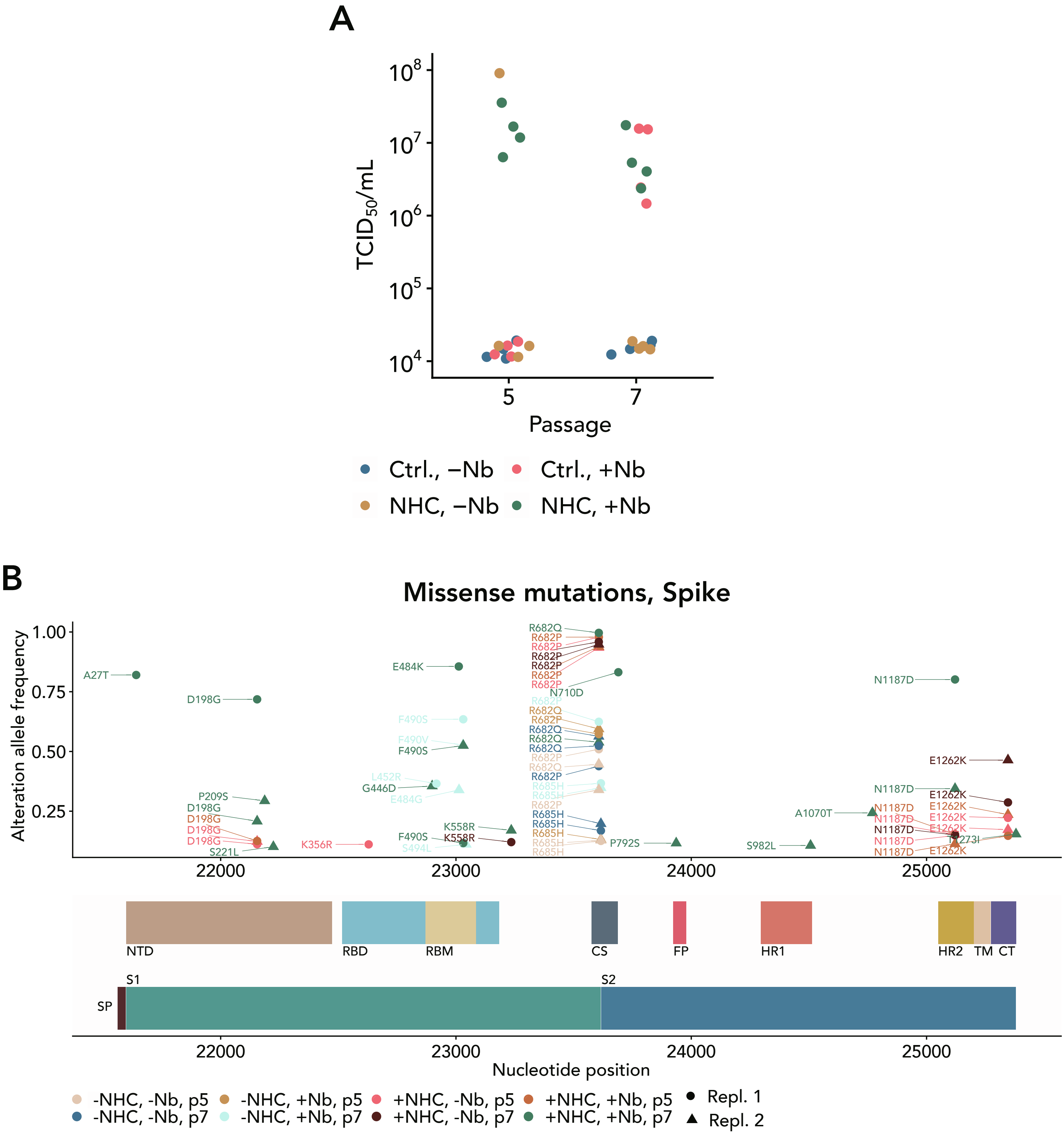

Figure S5, related to Figure 7

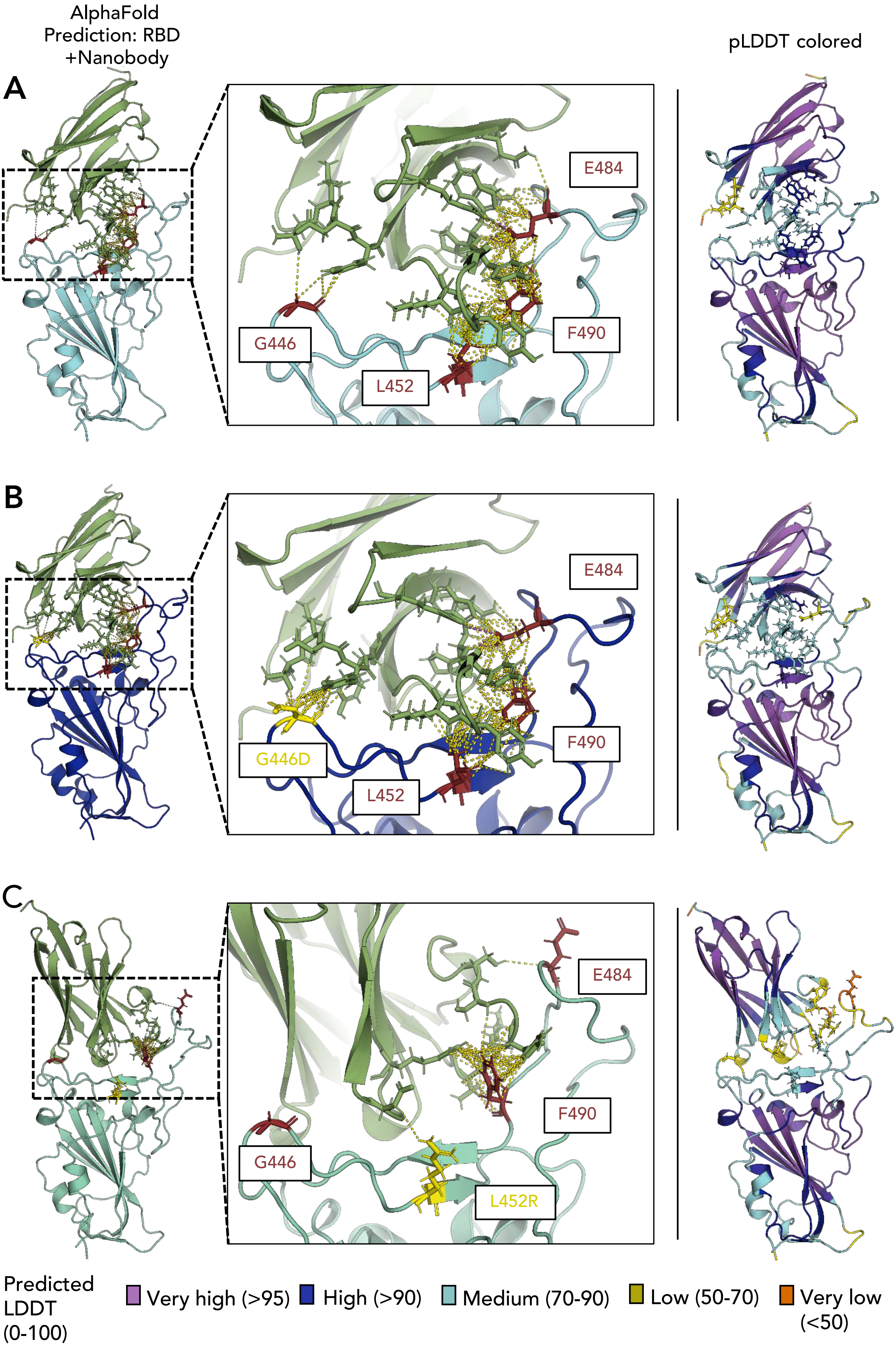

Figure S6, related to Figure 7

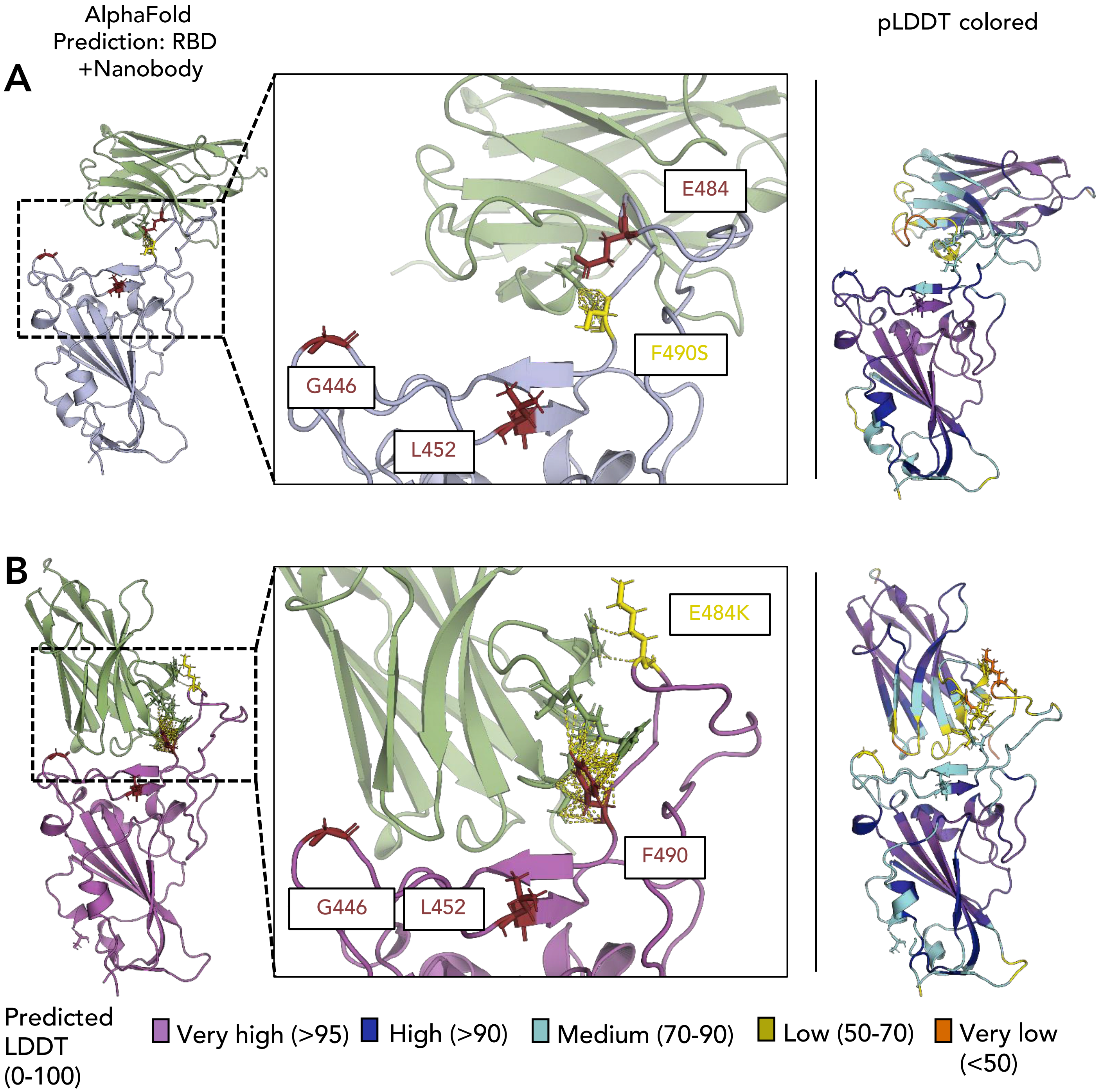

Figure S7, related to Figure 7

A

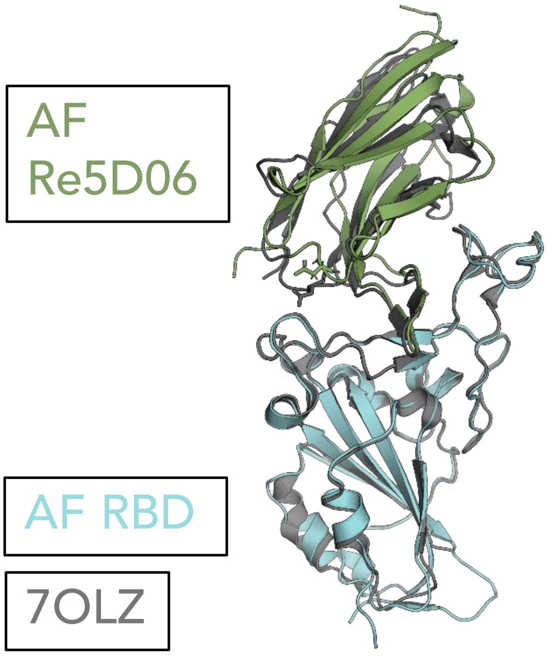

B

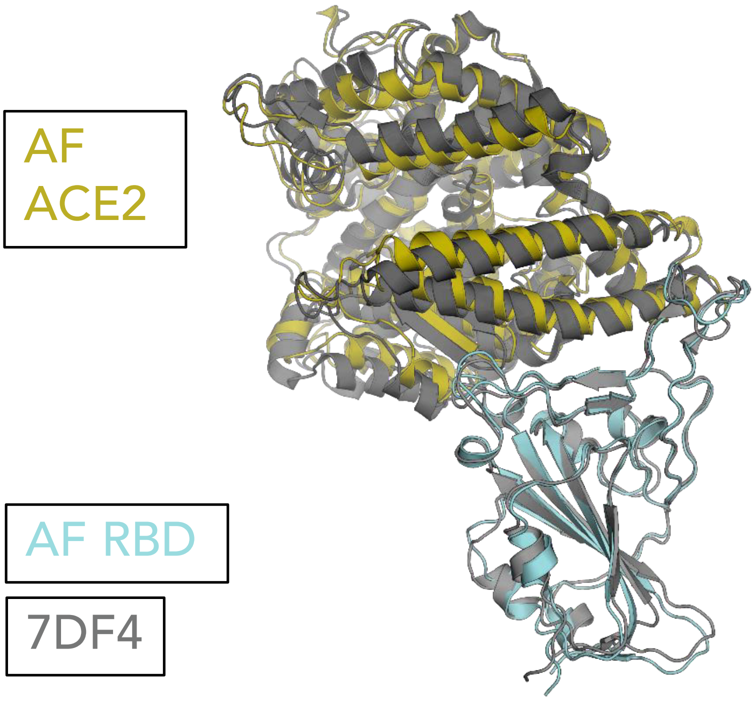

Figure S8, related to Figure 7

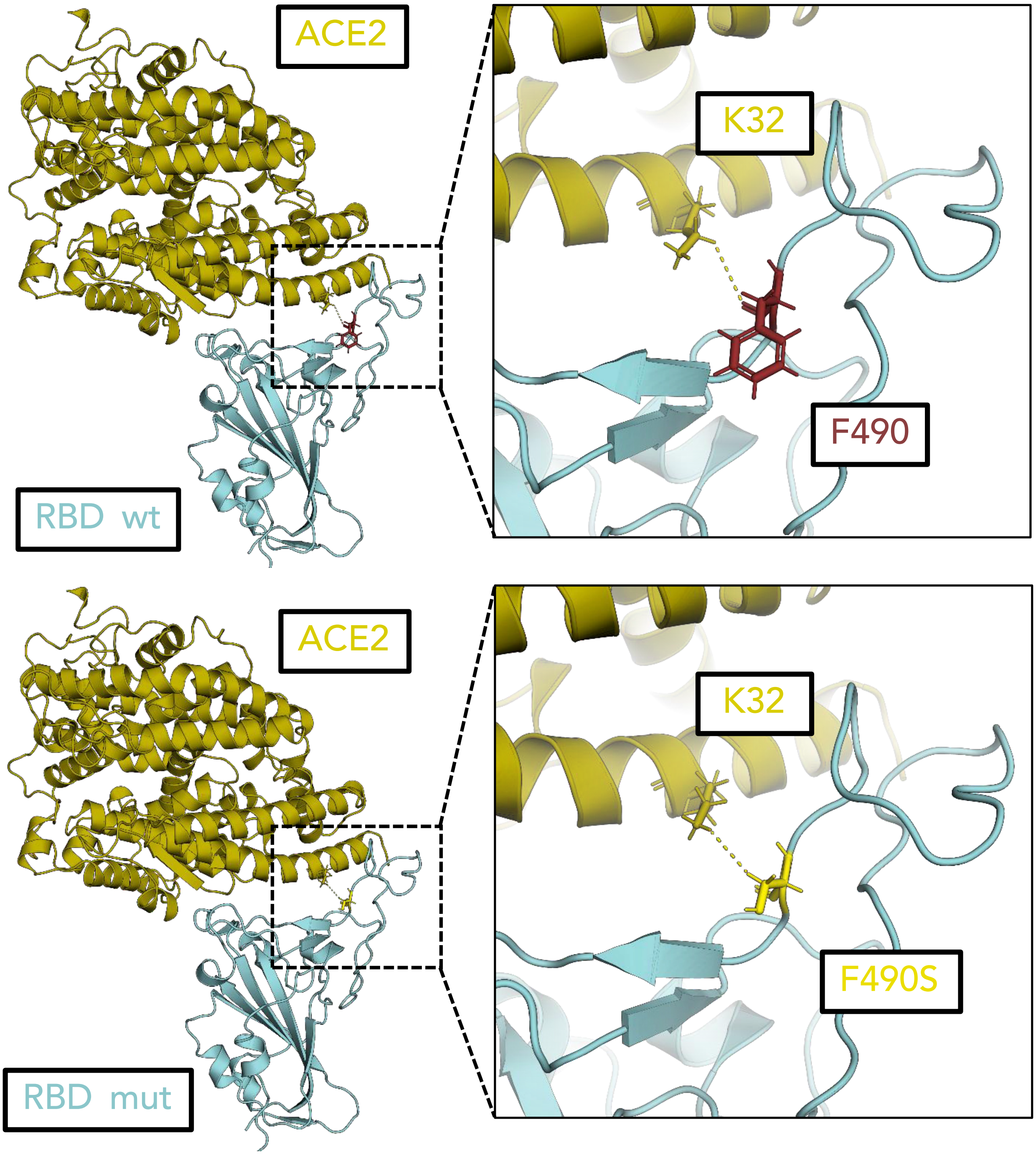

Supplement: Document S1. Figures S1–S8 [file mmc1.pdf]
